# Association of tiered restrictions and a second lockdown with COVID-19 deaths and hospital admissions in England: a modelling study

**DOI:** 10.1016/S1473-3099(20)30984-1

**Published:** 2021-04

**Authors:** Nicholas G Davies, Rosanna C Barnard, Christopher I Jarvis, Timothy W Russell, Malcolm G Semple, Mark Jit, W John Edmunds, Centre for Mathematical Modelling of Infectious Diseases COVID-19 Working Group, ISARIC4C investigators

**Affiliations:** a Department of Infectious Disease Epidemiology, London School of Hygiene & Tropical Medicine, London, UK; b National Institute for Health Research Health Protection Research Unit in Emerging and Zoonotic Infections, Institute of Infection, Veterinary and Ecological Sciences, Faculty of Health and Life Sciences, University of Liverpool, Liverpool, UK; c Department of Respiratory Medicine, Alder Hey Children's Hospital, Liverpool, UK

## Abstract

**Background:**

A second wave of COVID-19 cases in autumn, 2020, in England led to localised, tiered restrictions (so-called alert levels) and, subsequently, a second national lockdown. We examined the impact of these tiered restrictions, and alternatives for lockdown stringency, timing, and duration, on severe acute respiratory syndrome coronavirus 2 (SARS-CoV-2) transmission and hospital admissions and deaths from COVID-19.

**Methods:**

We fit an age-structured mathematical model of SARS-CoV-2 transmission to data on hospital admissions and hospital bed occupancy (ISARIC4C/COVID-19 Clinical Information Network, National Health Service [NHS] England), seroprevalence (Office for National Statistics, UK Biobank, REACT-2 study), virology (REACT-1 study), and deaths (Public Health England) across the seven NHS England regions from March 1, to Oct 13, 2020. We analysed mobility (Google Community Mobility) and social contact (CoMix study) data to estimate the effect of tiered restrictions implemented in England, and of lockdowns implemented in Northern Ireland and Wales, in October, 2020, and projected epidemiological scenarios for England up to March 31, 2021.

**Findings:**

We estimated a reduction in the effective reproduction number (*R*_t_) of 2% (95% credible interval [CrI] 0–4) for tier 2, 10% (6–14) for tier 3, 35% (30–41) for a Northern Ireland-stringency lockdown with schools closed, and 44% (37–49) for a Wales-stringency lockdown with schools closed. From Oct 1, 2020, to March 31, 2021, a projected COVID-19 epidemic without tiered restrictions or lockdown results in 280 000 (95% projection interval 274 000–287 000) hospital admissions and 58 500 (55 800–61 100) deaths. Tiered restrictions would reduce hospital admissions to 238 000 (231 000–245 000) and deaths to 48 600 (46 400–50 700). From Nov 5, 2020, a 4-week Wales-type lockdown with schools remaining open—similar to the lockdown measures announced in England in November, 2020—was projected to further reduce hospital admissions to 186 000 (179 000–193 000) and deaths to 36 800 (34 900–38 800). Closing schools was projected to further reduce hospital admissions to 157 000 (152 000–163 000) and deaths to 30 300 (29 000–31 900). A projected lockdown of greater than 4 weeks would reduce deaths but would bring diminishing returns in reducing peak pressure on hospital services. An earlier lockdown would have reduced deaths and hospitalisations in the short term, but would lead to a faster resurgence in cases after January, 2021. In a post-hoc analysis, we estimated that the second lockdown in England (Nov 5–Dec 2) reduced *R*_t_ by 22% (95% CrI 15–29), rather than the 32% (25–39) reduction estimated for a Wales-stringency lockdown with schools open.

**Interpretation:**

Lockdown measures outperform less stringent restrictions in reducing cumulative deaths. We projected that the lockdown policy announced to commence in England on Nov 5, with a similar stringency to the lockdown adopted in Wales, would reduce pressure on the health service and would be well timed to suppress deaths over the winter period, while allowing schools to remain open. Following completion of the analysis, we analysed new data from November, 2020, and found that despite similarities in policy, the second lockdown in England had a smaller impact on behaviour than did the second lockdown in Wales, resulting in more deaths and hospitalisations than we originally projected when focusing on a Wales-stringency scenario for the lockdown.

**Funding:**

Horizon 2020, UK Medical Research Council, and the National Institute for Health Research.

## Introduction

The UK saw its first wave of COVID-19 cases in spring, 2020. Following the imposition of a national lockdown on March 23, 2020, with residents required to stay at home except for accessing medical care, daily exercise, shopping for essentials, and essential work travel, COVID-19 cases, hospital admissions, and deaths subsided. A resurgence of COVID-19 cases began in the late summer after most restrictions had been lifted. By the end of October, large-scale, population-based studies in England suggested about 50 000–100 000 new infections were occurring every day.^1^, ^2^ This rise in infections resulted in pressure on health services, with 8822 confirmed COVID-19 cases in English hospitals on Oct 30^3^—about half the number observed during the previous peak in April—and increasing numbers of deaths. Evidence of substantial geographical heterogeneity began to emerge across England, with a national infection survey suggesting that in late October, around one in 45 people were infected in the northwest, compared with one in 200 in the southeast.^2^

**Research in context**

**Evidence before this study**

Numerous studies have modelled the relative effects of non-pharmaceutical interventions on severe acute respiratory syndrome coronavirus 2 (SARS-CoV-2) transmission. We searched PubMed, bioRxiv, and medRxiv from database inception to Nov 9, 2020, for English-language articles with the search terms (“COVID-19” OR “SARS-CoV-2” OR “coronavirus”) AND (“lockdown”) AND (“model”). This search returned 676 results, of which 23 were modelling studies that fit models to data and examined a second round of physical distancing restrictions, such as lockdowns or tiered restrictions. 19 of the 23 studies used a model to assess the impact of lockdowns, often on a national scale and occasionally regionally. The two studies most similar to our own considered tiered responses in China and so-called circuit breakers in the UK. However, typically, the length or stringency of the lockdown considered were not varied.

**Added value of this study**

This study builds upon the existing literature in several ways. First, mobility measures (Google Community Mobility) and contact survey data (CoMix study) were used to estimate behavioural responses following the introduction of tiered restrictions in England, the so-called firebreak lockdown in Wales, and the so-called circuit breaker lockdown in Northern Ireland. Second, the model was fit to multiple data sources to reconstruct the dynamics of the SARS-CoV-2 outbreak in England from March to October, 2020. Finally, policies for managing a second wave of COVID-19 cases are contrasted. Comparisons are made between a baseline scenario (ie, a counterfactual scenario with no tiered restrictions and no lockdown), implementation of tiered restrictions only, and implementation of tiered restrictions plus different-stringency lockdowns in England, with and without schools open. The effects of these scenarios on cumulative deaths, demand for hospital services, and time spent under restrictions are explored in relation to the type of intervention implemented and the duration and timing of lockdown interventions. Regional responses to different types, timings, and durations of interventions are also explored.

**Implications of all the available evidence**

Without the additional public health interventions adopted, the second wave is projected to be more severe than the first wave. The tiered restrictions introduced in October, 2020 (in particular tier 3), were projected to have had some effect in slowing transmission, but the addition of a temporary lockdown provided the strongest effect in reducing COVID-19 deaths and hospital admissions. Earlier lockdowns would have saved lives in the short term, but because substantial susceptibility would have remained in the population, they might have resulted in larger resurgences after January, 2021, requiring the introduction of further non-pharmaceutical interventions.

On Oct 12, the UK Government announced a programme of regionally differentiated physical distancing measures using a three-tiered approach, known as alert levels.^4^ By default, regions were placed into tier 1, the least restrictive tier, but could be moved into tiers 2 or 3 if incidence of infection increased. Regions in tier 1 had a 2200 h curfew for hospitality venues and restrictions on the number of individuals who could meet (the so-called rule of six). Tier 2 regions had additional restrictions on individuals from different households mixing, and residents were advised to avoid making unnecessary journeys. Regions in tier 3 had additional closures of hospitality and leisure venues, such as pubs and restaurants. In the weeks following the announcement, the UK Government placed several local authority districts—particularly in the north of England—into the highest restriction category, tier 3. Despite these measures, incidence continued to rise in all regions of England.^1^, ^2^ Consequently, on Oct 31, a new 4-week national lockdown for England was announced, beginning on Nov 5. The restrictions were broadly similar to those of the initial spring lockdown, but schools and universities were allowed to remain open. It remains unclear how effective the tiered restrictions were in reducing transmission and what additional reduction in transmission might have been accomplished by the second lockdown.

The other UK nations experienced similar resurgences in September, 2020, and in response to this, both Northern Ireland and Wales implemented time-limited lockdowns in mid-October. These differed in their stringencies, with the so-called firebreak measures in Wales being more comprehensive than the so-called circuit breaker measures in Northern Ireland. Both lockdowns were timed to coincide with the school half-term vacation period.

Here, we analyse mobility and contact survey data to estimate the impact of tiered restrictions in England and of the lockdowns in Northern Ireland and Wales on severe acute respiratory syndrome coronavirus 2 (SARS-CoV-2) transmission. We use this analysis to parameterise a mathematical model fit to multiple data sources to estimate the impact of tiered restrictions and alternative scenarios regarding the timing, duration, and stringency of extended physical distancing measures on hospital admissions and deaths due to COVID-19 in England.

## Methods

### Epidemiological model and fitting

We used a previously published dynamic compartmental model of SARS-CoV-2 transmission^5^, ^6^ stratified into 5-year age bands. The model was fitted using Markov chain Monte Carlo methods to data reported across the seven National Health Service (NHS) England regions on hospital admissions for COVID-19, hospital and intensive care unit (ICU) bed occupancy related to COVID-19, seroprevalence, PCR positivity, and deaths within 28 days of an individual's first positive SARS-CoV-2 test, which is used as the primary measure of COVID-19 mortality in the UK.^7^ Hospital admissions and occupancy data were provided by NHS England and deaths data by Public Health England (March 1–Oct 13). These data sources are unpublished and not public but are closely aligned with the UK Government COVID-19 Dashboard.^3^ Seroprevalence data were obtained from the Office for National Statistics COVID-19 Infection Survey (ONS-CIS; May 1–Sept 30),^2^ the UK Biobank (May 27–Aug 14),^8^ and the REACT-2 study (June 20–Sept 28),^1^ and PCR-positivity data were obtained from the REACT-1 study (May 1–Oct 5).^1^

The age-specific probability of ICU admission given hospital admission, as well as the distributions of lengths of stay in hospital and in the ICU, were estimated using individual-patient data in the COVID-19 Clinical Information Network (CO-CIN), which were collected from an ongoing study in patients with COVID-19 in the UK.^9^ Model-fitted distributions for the delays from infection to death, infection to hospital admission, and infection to ICU admission were also informed by CO-CIN data. The relative age-specific infection fatality risk was adopted from a global meta-analysis,^10^ and the relative age-specific infection hospitalisation risk was adopted from a study of the COVID-19 epidemic in France;^11^ given these relative age-specific rates, the overall infection fatality risk, infection hospitalisation risk, and probability of ICU admission given hospitalisation were inferred for each NHS England region during model fitting. The age-specific fatality risk among patients admitted to hospital decreased substantially over time according to CO-CIN data;^9^ therefore, we estimated this relative decrease during model fitting, assuming no further change in the infection fatality risk from September, 2020, onwards. A full description of fitted and non-fitted parameters is provided in the appendix (pp 8–10).

### Transmission rates and mobility indices

Because some of the most reliable indicators of infection—hospital admissions and deaths—lag substantially behind transmission rates, it is challenging to estimate the impact of policy and behavioural changes on SARS-CoV-2 transmission in real time. We measured the relationship between anonymised mobility data collected from smartphone users by Google Community Mobility^12^ and fine-grained social-contact survey data from the CoMix study,^13^ which has been collecting data on UK residents' daily interpersonal contacts since late March, 2020. This approach allowed us to use indirect but rapidly available mobility data to predict changes in transmission resulting from behavioural and policy changes over time. We used this approach both in fitting the model to policy changes over the first wave of the COVID-19 epidemic in England, and in estimating the impact of tiered restrictions in England and of lockdown interventions in Northern Ireland and Wales (appendix pp 1–5).

### Intervention scenarios

During Northern Ireland's so-called circuit breaker lockdown, non-essential retail remained open and so-called household bubbles of up to ten people from two households were allowed to mix. By contrast, during Wales' so-called firebreak lockdown, non-essential retail was closed and residents were advised to stay at home and were prohibited from mixing with individuals from outside their households. We therefore constructed a Northern Ireland-stringency lockdown scenario and a Wales-stringency lockdown scenario by applying the measured reduction in mobility in Northern Ireland and in Wales to England. Additionally, we modelled these scenarios either with schools closed or with schools open during lockdown. To simulate school closure, we reduced contact rates according to the age-specific proportion of contacts made at school,^14^ and further reduced all individuals' contacts by a multiplicative factor we estimated during model fitting that was associated with the reopening of schools in England in September (appendix pp 7–9). We also varied the duration and timing of lockdown interventions. Given that the UK Government opted for a 4-week lockdown in England from Nov 5, which was similar to the Welsh lockdown scenario without school closures, we focus on this scenario for our assessment of the impact of a second lockdown in England, examining other possibilities as sensitivity analyses. By default, we assume that recovery from SARS-CoV-2 infection confers lifelong immunity to reinfection, but we also explore a scenario with waning natural protection. Additionally, by default we assume that—except for changes imposed by restrictions—contact rates remained constant after the imposition of tiered restrictions on Oct 14; we also explored a scenario in which seasonal increases in contact patterns resulted in an increase in transmission over the winter period (appendix p 6). Throughout, we calculate 95% credible intervals (CrIs) and 95% projection intervals (PIs) as the 2·5th and 97·5th percentiles of the sampled posterior distribution for model outputs. The use of PIs emphasises that these intervals do not account for unforeseen changes in contact patterns and epidemiological parameters.

### Ethics

For CO-CIN data, ethical approval for data collection and analysis by ISARIC4C was given by the South Central-Oxford C Research Ethics Committee in England (reference 13/SC/0149) and the Scotland A Research Ethics Committee (reference 20/SS/0028). The ISARIC WHO CCP-UK study, which produced the CO-CIN data, is registered with the ISRCTN registry (ISRCTN66726260) and was designated an Urgent Public Health Research Study by the National Institute for Health Research.

### Role of the funding source

The funders of the study had no role in study design, data collection, data analysis, data interpretation, or writing of the report. NGD and RCB had full access to all the data in the study. The corresponding author had final responsibility for the decision to submit for publication.

## Results

**Figure 1:**
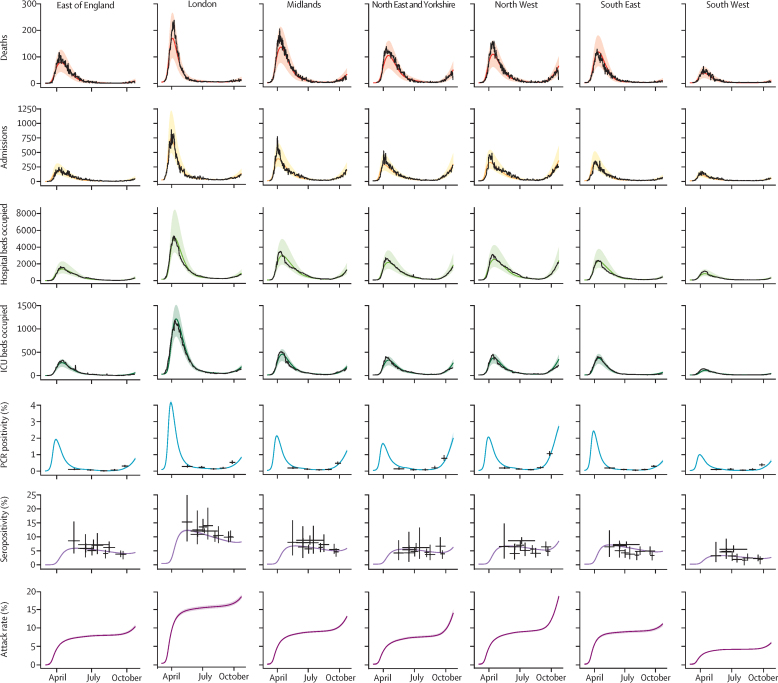
Model fit to NHS England region-specific data from March 1, to Oct 14, 2020 NHS England region-specific data on the number of COVID-19 deaths, hospital admissions, and occupied hospital and ICU beds; proportion of residents PCR positive and seropositive; and the regional attack rate (proportion ever infected). Black lines show reported data. Coloured lines and shaded areas show medians and 95% credible intervals from the fitted model. The crosses on the PCR positivity and seropositivity graphs show the time period over which the data were collected (horizontal lines) and the 95% CIs associated with the data (vertical lines). ICU=intensive care unit. NHS=National Health Service.

Our fitted model captures the observed dynamics of community transmission of SARS-CoV-2 during the first and second waves from March 1, to Oct 13, 2020 (figure 1), reproducing region-specific observed infections, seropositivity, deaths, hospital admissions, and ICU and hospital bed occupancies. In addition, the model was capable of accurately forecasting the changes in numbers of deaths, hospital admissions, and hospital beds occupied during the autumn period, although it overestimated ICU occupancy due to a sharp decline in the proportion of patients admitted to hospital who were admitted to the ICU after mid-September (appendix pp 12–16).

Under our base-case assumption of no waning immunity and no seasonal increase in contacts, and without the imposition of tiered restrictions or any other lockdown intervention, the model suggests that hospital admissions would peak in the North West around mid-November, with other regions peaking somewhat later over the winter period (appendix p 22). By March 31, 2021, around 35–46% of the population in each region is expected to have been infected (appendix p 34). Under this baseline scenario, from Oct 1, 2020, to March 31, 2021, the epidemic is projected to result in 280 000 (95% PI 274 000–287 000) hospital admissions and 58 500 (55 800–61 100) deaths, with a peak ICU occupancy of 5000 (4840–5170) beds (table 1). For the first wave (up to Sept 30), our fitted model estimates there were 127 000 (125 000–128 000) hospital admissions and 36 900 (36 200–37 500) deaths, and that the peak ICU occupancy was 3090 (3020–3130) beds. Therefore, the expected scale of the second wave, without any interventions, is larger than the first on all three of these key metrics. Crucially, this baseline scenario for the second wave is not equivalent to a completely unmitigated epidemic, as social contacts have not returned to their prepandemic rates in England (appendix pp 1–2), and the incidence of infection is also blunted by immunity acquired during the first wave. There are, however, expected to be considerable differences between regions in the epidemic burden, with the greatest number of admissions and deaths projected for the Midlands, North East and Yorkshire, and North West regions (appendix p 28).

**Table 1:** Model projections in England for the period of Oct 1, 2020, to March 31, 2021

	**Baseline scenario**	**Tiers only**	**Northern Ireland-type lockdown**		**Wales-type lockdown**	
			Schools open	Schools closed	Schools open	Schools closed
Hospital admissions	280 000 (274 000–287 000)	238 000 (231 000–245 000)	206 000 (199 000–213 000)	177 000 (171 000–181 000)	186 000 (179 000–193 000)	157 000 (152 000–163 000)
Deaths	58 500 (55 800–61 100)	48 600 (46 400–50 700)	41 500 (39 600–43 400)	34 900 (33 500–36 700)	36 800 (34 900–38 800)	30 300 (29 000–31 900)
Peak ICU occupancy, %*	168% (162–174)	131% (128–135)	96% (93–102)	88% (85–91)	90% (85–94)	87% (83–91)
Peak ICU occupancy, beds	5000 (4840–5170)	3900 (3800–4010)	2870 (2760–3040)	2610 (2520–2720)	2670 (2540–2810)	2590 (2480–2710)
Time in tier 2, weeks†	0 (0–0)	11·4 (10·0–12·7)	12·0 (10·8–13·3)	8·5 (8·2–8·8)	9·0 (8·3–9·6)	7·5 (7·1–7·8)
Time in tier 3, weeks†	0 (0–0)	4·00 (2·96–5·03)	0·48 (0·37–0·58)	0·47 (0·36–0·57)	0·47 (0·35–0·57)	0·47 (0·35–0·58)
Time in lockdown, weeks†	0 (0–0)	0 (0–0)	3·9 (3·9–3·9)	3·9 (3·9–3·9)	3·9 (3·9–3·9)	3·9 (3·9–3·9)
Time spent under high ICU occupancy, weeks†	14·8 (14·7–15·0)	14·6 (14·3–14·9)	13·7 (12·9–14·7)	9·5 (9·1–10·0)	11·3 (10·3–12·7)	7·9 (7·5–8·4)

Medians and 95% projection intervals are shown. Weeks of high ICU occupancy are calculated by measuring the number of weeks in each region where ICU occupancy is 50% or greater than the peak occupancy during the first wave. Lockdowns are assumed to run from Nov 5 to Dec 2, 2020 (eg, 3·9 weeks), inclusively. ICU=intensive care unit.

* Relative to the peak ICU occupancy in March–May, 2020 (the first wave).

† The underlying quantity is the population-weighted mean time spent living under restrictions across all seven National Health Service England regions.

Our analysis of mobility indicators suggests that tier 3 restrictions are associated with a substantially greater reduction in mobility than are tier 2 restrictions. In turn, both lockdowns are associated with a greater reduction in mobility than are tier 3 restrictions, with the firebreak lockdown in Wales having a substantially greater effect than the circuit breaker in Northern Ireland (appendix pp 2–5). In turn, these reductions in mobility are estimated to reduce the effective reproduction number (*R*_t_) by 2% (95% CrI 0–4) for tier 2, 10% (6–14) for tier 3, 35% (30–41) for a Northern Ireland-stringency lockdown with schools closed, and 44% (37–49) for a Wales-stringency lockdown with schools closed (table 2). When we introduce tiered restrictions into our model on Oct 14, 2020, the projected number of hospital admissions from Oct 1, 2020, to March 31, 2021, decreases to 238 000 (95% PI 231 000–245 000), of deaths to 48 600 (46 400–50 700), and of number of beds occupied at peak ICU occupancy to 3900 (3800–4010; table 1).

**Table 2:** Estimated effect of tiered restrictions and lockdowns on *R*_t_ of SARS-CoV-2 in England

		**Reduction in *R*_t_**
Tier 2		2% (0–4)
Tier 3		10% (6–14)
Northern Ireland-type lockdown		
	Schools closed	35% (30–41)
	Schools open	22% (15–27)
Wales-type lockdown		
	Schools closed	44% (37–49)
	Schools open	32% (25–39)
England lockdown		
	Schools closed	36% (29–42)
	Schools open	22% (15–29)

Data are % (95% credible interval). The reduction in *R*_t_ is relative to immediately before the control measure was introduced. *R*_t_=effective reproduction number. SARS-CoV-2=severe acute respiratory syndrome coronavirus 2.

**Figure 2:**
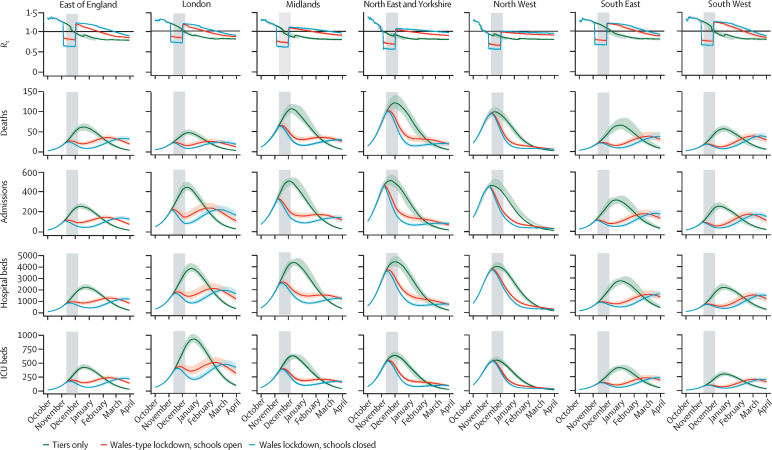
Projected impact of a Wales-type lockdown in NHS England regions *R*_t_, the daily number of deaths and hospital admissions, and the daily number of occupied hospital and ICU beds are compared across seven NHS England regions for three different scenarios: tiered restrictions only and a Wales-type lockdown with and without schools open. Lockdowns extend from Nov 5, to Dec 2, 2020 (indicated by grey shading on the graphs). Lines and green, red, or blue shaded ribbons indicate median and 95% projection intervals, respectively. Step changes in *R*_t_ show the introduction or relaxation of tiered restrictions and lockdown measures. ICU=intensive care unit. NHS=National Health Service. *R*_t_=effective reproduction number.

The model projects a reduction in transmission across all NHS England regions following the introduction of a 4-week Wales-type lockdown (figure 2), with the closure of schools resulting in additional reductions in transmission. *R*_t_ is suppressed to below one during lockdown periods. In most regions, following the lockdown period, *R*_t_ initially increases above one before reducing over time. This rebound occurs because there is insufficient immunity in the population, and so as restrictions are eased, transmission increases. By contrast, in the most heavily affected regions (ie, the North West), the easing of lockdown is not expected to result in a rebound of infections as accumulated population immunity retains *R*_t_ below one. We observe similar results (including reduction of *R*_t_ below one in all regions) upon the introduction of a Northern Ireland-type lockdown (appendix p 23), but with weaker effects. Region-specific effects on hospital admissions, deaths, ICU burden, and length of time spent under different measures for different lockdown scenarios are shown in the appendix (pp 30–33). The model predicts that the North East and Yorkshire, North West, and South West regions exceed the peak ICU occupancy observed during the first wave for all four lockdown scenarios considered.

For the tiers-only scenarios, *R*_t_ decreases over time and remains below the levels expected with the introduction of a lockdown (figure 2; appendix p 23). This difference is due to greater depletion of susceptible individuals under tiered restrictions than with a lockdown; lockdowns reduce infections in the short term and therefore result in less population immunity.

**Figure 3:**
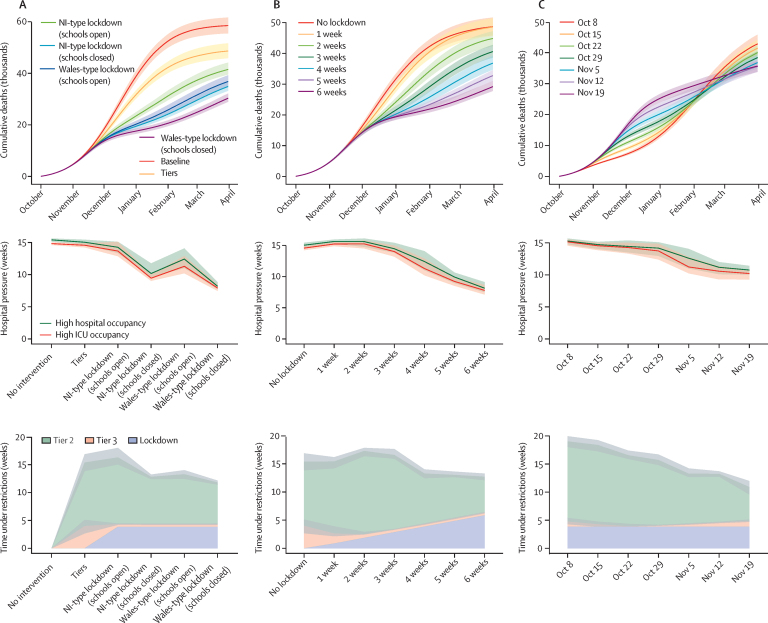
Projected impact of altering the type of intervention or duration or timing of lockdown on cumulative deaths, pressure on hospitals, and time spent living under restrictions (A) Impact of altering the type of intervention. Baseline refers to a counterfactual scenario with no tiered restrictions and no lockdown. Note that the lines for NI with and without schools closed overlap in the top graph. (B) Impact of altering the duration of lockdown. Graphs show the effects of introducing different lengths of a Wales-type lockdown (with schools open) in England on Nov 5, assuming tiered restrictions are already in place. (C) Impact of altering the timing of lockdown. Graphs show the effects of varying the time a 4-week, Wales-type lockdown (with schools open) is introduced in England, starting from up to 4 weeks before to 2 weeks after Nov 5. Hospital pressure was defined as the population-weighted mean number of weeks that an National Health Service region's hospital or ICU bed occupancy exceeded 50% of the peak occupancy for that region during the first wave of COVID-19 in England. All graphs show medians, with shaded regions defining 95% projection intervals. ICU=intensive care unit. NI=Northern Ireland.

Lockdown measures consistently outperform the baseline and tiered restrictions in reducing cumulative deaths over the time period considered (figure 3A). The higher the stringency of the lockdown and the longer the duration, the greater reduction in deaths (figure 3A, B). To substantially reduce pressure on health services, at least a Northern Ireland-type lockdown with schools closed (or a Wales-type lockdown with schools open) is required. Under a Wales-type lockdown with schools open, longer lockdown lengths result in lower numbers of cumulative deaths over time as well as reduced hospital pressure (figure 3B). Lockdowns also reduce the median time spent living under tiers 2 and 3 restrictions (figure 3A, B), illustrating that these interventions trade off against each other.

When a lockdown intervention is introduced earlier, the rise in deaths is suppressed sooner (figure 3C). However, by the end of March, 2021, we observe that scenarios with an earlier lockdown reach a higher cumulative number of deaths. This larger burden is because earlier lockdowns result in longer periods of inflated transmission following the end of the periods of restrictions. In reality, we expect that additional interventions would be introduced before this level of transmission is reached.

The best timing of a single 4-week lockdown, in terms of reductions in deaths and hospital pressure over the period considered, appears to be around early to mid-November, 2020 (figure 3C). This result is predicated upon only one 4-week lockdown being introduced, and takes as given the high level of SARS-CoV-2 transmission observed in England in autumn 2020. The effects on deaths, hospital admissions, cases, infections, hospital burden, ICU burden, and the median time spent under restrictions for different intervention strategies and lockdown durations and timings were also explored (figure 3), and substantial variation was observed among regions, with the North West, North East and Yorkshire, and Midlands regions experiencing the greatest burdens (appendix p 24).

Table 3 shows the results of sensitivity analyses that include waning immunity, seasonal increases in contact patterns, or both, for the Wales-type lockdown without school closures (closest to the measures adopted in England) and for the tiers-only scenario. Both waning immunity and increases in mixing due to seasonal factors are expected to exacerbate the second wave. For instance, for the tiers-only scenario, seasonality is expected to increase demand for hospital beds and deaths by about 20%, waning immunity by about 25%, and both waning immunity and seasonality by about 50%. Increasing transmission over the winter period as a result of either or both of these factors diminishes the impact of the temporary lockdown, both in absolute and relative terms. For instance, under the baseline scenario, the 4-week November lockdown was expected to reduce hospital admissions by about 52 000 between October, 2020, and March 2021, a 21% reduction. However, if both waning immunity and seasonality occur, introducing the same temporary lockdown would only be expected to reduce hospital admissions by about 31 000 over the same time period (a 9% reduction), as the rebound in infections after the lockdown would be more rapid under this scenario (appendix pp 25–27).

**Table 3:** Sensitivity analysis for scenarios with a seasonal increase in contact rates, waning immunity, or both

	**Tiers only**	**Lockdown**	**Tiers only plus seasonality**	**Lockdown plus seasonality**	**Tiers only plus waning**	**Lockdown plus waning**	**Tiers only plus seasonality and waning**	**Lockdown plus seasonality and waning**
Hospital admissions	238 000 (231 000–245 000)	186 000 (179 000–193 000)	283 000 (274 000–289 000)	247 000 (240 000–253 000)	297 000 (288 000–304 000)	260 000 (252 000–267 000)	355 000 (345 000–364 000)	324 000 (316 000–333 000)
Deaths	48 600 (46 400–50 700)	36 800 (34 900–38 800)	58 100 (55 400–61 100)	49 900 (47 300–51 900)	59 200 (57 000–61 600)	50 200 (48 200–52 500)	72 300 (69 300–76 100)	64 100 (61 600–67 400)
Peak ICU occupancy, %*	131% (128–135)	90% (85–94)	166% (161–173)	122% (119–127)	148% (143–153)	120% (117–124)	193% (187–203)	152% (148–158)
Peak ICU occupancy, beds	3900 (3800–4010)	2670 (2540–2810)	4950 (4780–5150)	3640 (3540–3790)	4400 (4250–4550)	3570 (3470–3680)	5760 (5570–6050)	4510 (4400–4690)
Time in tier 2, weeks†	11·4 (10·0–12·7)	9·0 (8·3–9·6)	7·0 (6·8–7·8)	12·9 (11·3–14·0)	11·6 (10·3–12·8)	14·9 (14·1–15·7)	10·1 (9·5–10·3)	13·2 (12·6–14·3)
Time in tier 3, weeks†	4·00 (2·96–5·03)	0·47 (0·35–0·57)	8·00 (7·29–8·00)	1·51 (0·91–2·27)	6·26 (5·64–6·99)	1·50 (1·06–2·25)	8·67 (8·67–8·67)	5·19 (4·37–5·70)
Time in lockdown, weeks†	0 (0–0)	3·86 (3·86–3·86)	0 (0–0)	3·86 (3·86–3·86)	0 (0–0)	3·86 (3·86–3·86)	0 (0–0)	3·86 (3·86–3·86)
Time spent under high ICU occupancy, weeks†	14·6 (14·3–14·9)	11·3 (10·3–12·7)	15·1 (14·9–15·4)	16·8 (16·4–17·5)	17·6 (17·3–18·0)	18·4 (17·7–19·1)	18·2 (17·9–18·6)	20·7 (20·0–21·3)

Medians and 95% projection intervals are shown. Burdens are summed over the period from Oct 1, 2020, to March 31, 2021. Here, the lockdown scenario uses the assumption of a Wales-type lockdown with schools open. Lockdowns are assumed to run from Nov 5, to Dec 2, 2020, inclusively. Seasonal contact patterns and waning protection from reinfection take effect on Oct 1, 2020. ICU=intensive care unit.

* Relative to the peak ICU occupancy in March–May, 2020 (the first wave).

† The underlying quantity is the population-weighted mean time spent living under restrictions across all seven National Health Service England regions.

These analyses were originally conducted during late October and early November, 2020, when the decisions over a lockdown in England were being made. At the end of November, we assessed the actual impact of the lockdown in England on observed mobility and used these estimates to update our projections. The lockdown in England had an effect on mobility that was intermediate between the effects of the lockdowns in Wales and Northern Ireland (table 2; appendix pp 4–5). Qualitatively, the projected impact of the 4-week lockdown in England on estimated cumulative deaths, hospital pressure, and time spent under different restrictions is similar to our base-case analysis, but the lower impact of the lockdown on behaviour means that the distinction between the effects of alternative policies in terms of timing and duration is less marked than with the base-case scenario (appendix pp 7, 11–12).

## Discussion

Without additional restrictions, the second wave of COVID-19 in England is projected to be more severe than the first wave in terms of hospital admissions and deaths. Tiered restrictions, and in particular the most stringent tier 3, probably helped to slow transmission, although these restrictions have a much lesser effect on reducing hospital admissions and deaths than do lockdown scenarios. We projected that a 4-week lockdown intervention would probably have a strong but temporary effect, reducing *R*_t_ to well below one during the lockdown period, with sustained reductions in cases, deaths, and hospital admissions for several months afterwards. After easement of the lockdown, we do not expect a large surge in cases if tiered restrictions remain in place, because in most NHS England regions we project there will be sufficient depletion of susceptible individuals—given transmission and contact rates as of early December, 2020—to keep *R*_t_ below, or close to, one. However, outbreaks could still occur, particularly in areas with previously low incidence. If there is a seasonal increase in transmission during winter, substantial waning immunity, or a relaxation in control measures including tiered restrictions, there could be a larger resurgence in transmission.

Among the lockdown scenarios we considered, the timing of lockdown as enacted in England (Nov 5) is roughly consistent with the largest reduction in deaths and least pressure on health services. An earlier lockdown is projected to have saved more lives up to the end of January, 2021, but might have resulted in a larger resurgence in February and March, 2021, in the absence of additional measures (figure 3). These conclusions are broadly in line with other studies considering the impact of tiered restrictions and lockdown interventions. A network-based study considering the effect of tiered restrictions in China concluded that later implementation of lockdowns and physical distancing measures significantly increases the total number of infections.^15^ Another study looking at short-term, so-called circuit breaker interventions in the UK found that such interventions have the biggest impact when the growth rate is low.^16^ The authors concluded that such interventions are not long-term solutions but can buy time to improve other control measures, such as testing, contact tracing and isolation, or introduction of a vaccine.

We arrived at our conclusions by jointly fitting our age-structured transmission model of SARS-CoV-2 to the following data sources: observed hospital admissions, hospital and ICU bed occupancy, seroprevalence, PCR positivity, and deaths. The model fit well to these data streams and predicted the time course of hospital admissions and deaths accurately over the course of the autumn, giving some confidence in the results shown here (appendix pp 12–16, 21). The inability of the model to accurately predict the level of tiered restrictions that two regions were placed under by the end of October, 2020 (appendix p 13), emphasises the difficulty of predicting political decisions. Projecting the epidemic over long time frames is inherently uncertain for many reasons, including that new interventions (such as mass screening or vaccination) might be introduced. Accordingly, these results should be taken as indicative of what might be expected if current policies remain in place, with a return to tiered restrictions after lockdown, rather than forecasts or predictions. For these reasons, we also chose the end of March, 2021, as the longest timescale to model. Indeed, the roll-out of vaccines is now underway.^17^ The availability of a vaccine might foreshorten the appropriate time frame for this analysis, placing further weight on the importance of prompt action to curb the second wave, although it will still be several months before vaccination starts to have a population-level impact.

Our model is subject to certain limitations and uncertainties, a number of which have previously been discussed in detail.^5^, ^6^ First, we do not consider the implementation of any further interventions after the lockdown periods considered, aside from a continuation of tiered restrictions at the level imposed before the lockdown. Second, for the majority of scenarios considered, we have assumed that once individuals have been infected with SARS-CoV-2 and recovered, their immunity is maintained over the time frames modelled. There is emerging evidence to suggest that reinfection with SARS-CoV-2 is possible, although rare.^18^ At present it is unclear how widespread reinfection events are, on what timescale these reinfection events might occur, and whether reinfection results in greater or lesser severity of disease.^19^ Given that we have projected that all regions of England are likely to have an *R*_t_ close to the critical threshold of one through to winter and early spring, 2021, relatively small changes in the level of natural immunity could have a substantial impact on predictions.

Although not the main focus of the work, we explored the effects of seasonal contact patterns and waning protection from reinfection, both of which worsen outcomes (table 3). Changes in behaviour are likely to occur over the time frames that we are modelling, particularly over the Christmas period. Behavioural changes are difficult to predict, and it is possible that there will be a return to more typical behaviours after the lockdown, or indeed a continuation of cautious behaviours, as was observed after the spring lockdown ended. We have not attempted to capture these possible changes; an improved understanding of how behaviours might alter in light of changes in risk and government advice is urgently needed to improve the longer-term accuracy of modelling studies. Moreover, it should be stressed that this model only considers morbidity and mortality directly related to COVID-19 (and might even underestimate COVID-19-related mortality due to the use of the standard definition for deaths related to COVID-19 within the UK—ie, death within 28 days of an individual's first COVID-19 test). There are a range of other COVID-19-related outcomes related to short-term illness and long-term sequelae that we do not consider here. There are also many indirect health effects that can result from disruption to health services, for which our measures of health service pressure are only a rough proxy. Moreover, there are multiple social, psychological, economic, and (for children) developmental costs and loss of educational opportunity, both in the short and longer term, resulting from interventions. We focus on direct epidemiological implications for COVID-19 and do not attempt to measure wider effects here, but we acknowledge that they must be taken into account when deciding on a course of action. Finally, our analysis was conducted before the emergence of the novel SARS-CoV-2 variant of concern 202012/01 in England, which might lead to increased transmissibility over the winter period.^20^ The potential for the emergence of more virulent strains of SARS-CoV-2 emphasises the need to quantify the effect of alternative non-pharmaceutical control measures, so that effective evidence-based policy can be made in response to developing circumstances. Crucially, we evaluated the efficacy of control measures by estimating their effect on mobility and contact patterns rather than on the incidence of infection. This means that our assessment of the effect of control measures on mobility in England (appendix pp 1–5) is not confounded by any increase in transmission owing to the emergence of the new variant.

Faced with rising COVID-19 cases and resulting pressure on health systems, countries across Europe have tried to adopt measures that maximise the suppression of transmission while minimising social and economic harms. Many have chosen to reintroduce strict measures such as lockdowns. In England, the government introduced a second national lockdown starting on Nov 5, 2020. We estimate that this lockdown will reduce COVID-19 deaths and ease the pressure on health services over the winter of 2020–21. More stringent or lengthy interventions could reduce deaths and hospital pressure further, but these benefits need to be weighed against the heavier social and economic costs associated with stricter measures.

## Data sharing

All analysis code and data are available at https://github.com/nicholasdavies/covid-tiers. The original report submitted to policy makers on Oct 31, 2020, has been archived at https://cmmid.github.io/topics/covid19/uk-tiers-2nd-lockdown.html.
