# Supplementary material for: Association of tiered restrictions and a second lockdown with COVID-19 deaths and hospital admissions in England: a modelling study
Source: Lancet Infect Dis. 2021 Apr;21(4):482–92. doi: 10.1016/S1473-3099(20)30984-1 (PMC7758181; doi:10.1016/S1473-3099(20)30984-1)
Supplement: Supplementary appendix [file mmc1.pdf]

THE LANCET
Infectious Diseases

Supplementary appendix

This appendix formed part of the original submission and has been peer reviewed. We post it as supplied by the authors.

Supplement to: Davies NG, Barnard RC, Jarvis CI, et al. Association of tiered restrictions and a second lockdown with COVID-19 deaths and hospital admissions in England: a modelling study. *Lancet Infect Dis* 2020; published online Dec 23. https://doi.org/10.1016/S1473-3099(20)30984-1.

Appendix

Mobility and contact rates

We analysed the relationship between mobility indices for England from the Google Community Mobility report[1] and surveyed social contact rates from the POLYMOD study[2] and the CoMix study[3].

Specifically, we conducted an exploratory analysis to determine which mobility indicators, if any, could be used to predict social contact rates—in home, work, school, or other settings—on a given day. We could not identify a convincing relationship between mobility indices and interpersonal contact in the home or school settings, which were better predicted by simple step functions following the first imposition of lockdown measures in England and school closures. We did, however, identify a clear relationship between workplace contacts reported in CoMix and the "workplace visits" indicator of Google Mobility, and between "other" contacts in social contact surveys and the "retail and recreation", "grocery and pharmacy", and "transit stations" indicators of Google Mobility. We characterised these relationships by fitting generalised additive models (GAM) of workplace and of "other" contacts to Google Mobility indicators using the R package mgcv[4], using workplace visits in Google Mobility as the predictor for workplace contacts in CoMix and using a weighted average of "retail and recreation", "grocery and pharmacy" and "transit station" visits in Google Mobility as the predictor for "other" contacts in CoMix. The optimal weighting of these three indicators was identified by optimising coefficients over the 3-simplex (i.e., 3 non-negative numbers summing to 1) to achieve the maximum deviance explained in the generalised additive model. Baseline levels of contact (those applying in the model prior to the first lockdown in March) were taken from the POLYMOD survey that collected contact data from the UK in 2006[2]. The changes in home and school contacts as a result of lockdown and school closures, and the relationship between workplace and other contacts and Google Mobility data is shown in **Fig. S1**.

We found that the average daily number of home contacts was well described by a step function transitioning from an average of 3.89 home contacts before March 23rd (the date of the first lockdown in the UK) to an average of 1.54 home contacts after March 23rd, with no evidence for substantial changes in the daily number of home contacts following an initial reduction in home contacts coinciding with the first lockdown in the UK (Fig. S1a). The average daily number of school contacts was well described by a step function transitioning from an average of 5.67 school contacts among individuals 18 or younger while schools were open, and zero school contacts when schools were closed (Fig. S1b). The average daily number of work contacts was well approximated by an approximately linear function of the number of "workplace" visits in the Google Mobility dataset, averaged across regions of England, extending from zero workplace contacts when workplace visits were at 23% of the baseline rate or lower, to 2.65 workplace contacts when workplace visits were at 100% of the baseline rate (Fig. S1c). Finally, the average daily number of "other" contacts was well approximated by a curved function of a weighted combination of transit station visits (44.5%), retail and recreation visits (34.5%), and grocery and pharmacy visits (21.0%; Fig. S1d). The particular functions for work and other contacts were obtained using thin-plate regression using the mgcv R package[5], while the coefficients used to weight transit station, retail and recreation, and

grocery and pharmacy visits were obtained by optimising a thin-plate regression model for maximum deviance explained over the 3-simplex of potential coefficients summing to 100%.

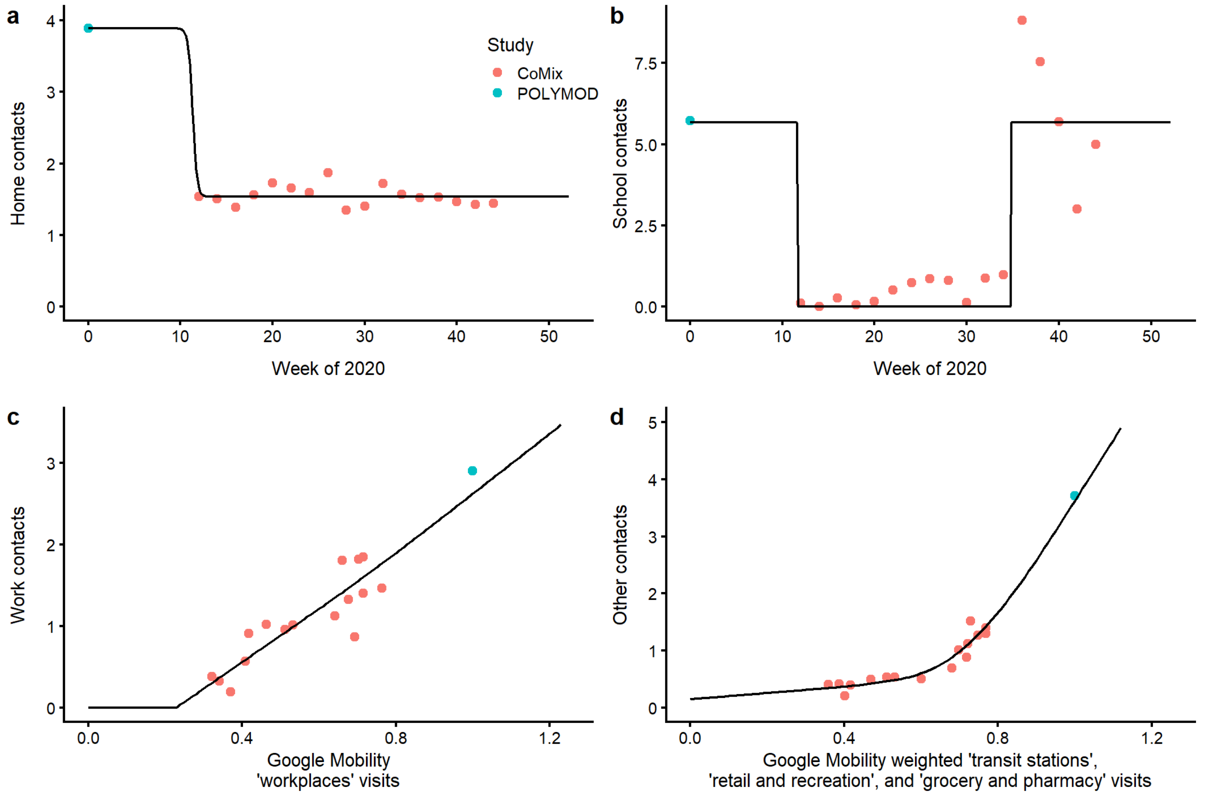

Fig. S1. Analysis of social contact rates during the SARS-CoV-2 epidemic in England. We illustrate the results of our analysis of social contact rates using the POLYMOD and CoMix contact surveys in the UK, using either the calendar date (a, b) or Google Community Mobility indices (c, d) as predictors. Shown are the obtained functions describing (a) home contacts (all individuals), (b) school contacts (among individuals 18 years of age or under), (c) work contacts (among individuals 18 to 65 years old), and (d) other contacts (among all individuals). Each point for CoMix represents an aggregated two-week period. For school contacts, some survey respondents in weeks 36–39 were reporting contact with all members of their class instead of conversational/physical contacts only, resulting in an illusory spike in contact rates at the beginning of the school year; the half-term break in week 43 resulted in fewer school contacts for the week 42–43 data point.

Impact of tiered restrictions (Alert Levels 1–3)

On October 14, 2020, the UK government announced a system of “Alert Level” tiered restrictions to be applied at the local authority level depending upon the local burden of disease. To estimate the impact of these tiered restrictions, we analysed how Google Community Mobility indicators in each region under restrictions (i.e., those in Tier 2 or Tier 3) changed relative to the regions without additional restrictions (i.e., those in Tier 1). We began by isolating Google Community Mobility indicators as a time series extending from 1 September to 27 October 2020, the most recent data available relating to tiered restrictions at the time of the analysis. There were 2,112 trend lines in total, representing six indicators for residential, workplace, park, grocery and pharmacy, retail and recreation, and transit station

visits for each of 352 local regions tracked by Google Community Mobility in England. There was a substantial day of week effect in all trend lines, which we removed by fitting a generalised additive model to estimate the weekday effect using the mgcv R package, fitting a cyclic spline with 7 knots — one for each day of the week — to each trend line (i.e., for each region and indicator available), and then subtracting this effect from each trendline. We then estimated a national trend for each of the six indicators using a generalised additive model with thin-plate spline regression and subtracted this national trend from the trend lines with weekday effects removed. Finally, for each trend line, we estimated a "baseline" value by taking the mean mobility index over the last seven days of data prior to the start of tiered restrictions, i.e. from 7 to 13 October 2020. We estimated the effect of each tier on mobility indices by comparing, for each region and mobility indicator, the difference between the baseline value and the mean mobility index over all days for which the region was under a given tier. This yielded 299 point estimates of the impact of Tier 1 relative to baseline, 104 point estimates of the impact of Tier 2 relative to baseline, and 35 estimates of the impact of Tier 3 relative to baseline, for each of the six mobility indicators (Fig S2). We used the mean difference between Tier 2 and Tier 1 and the mean difference between Tier 3 and Tier 1 in these point estimates as a measure of the impact of Tiers 2 and 3 on mobility.

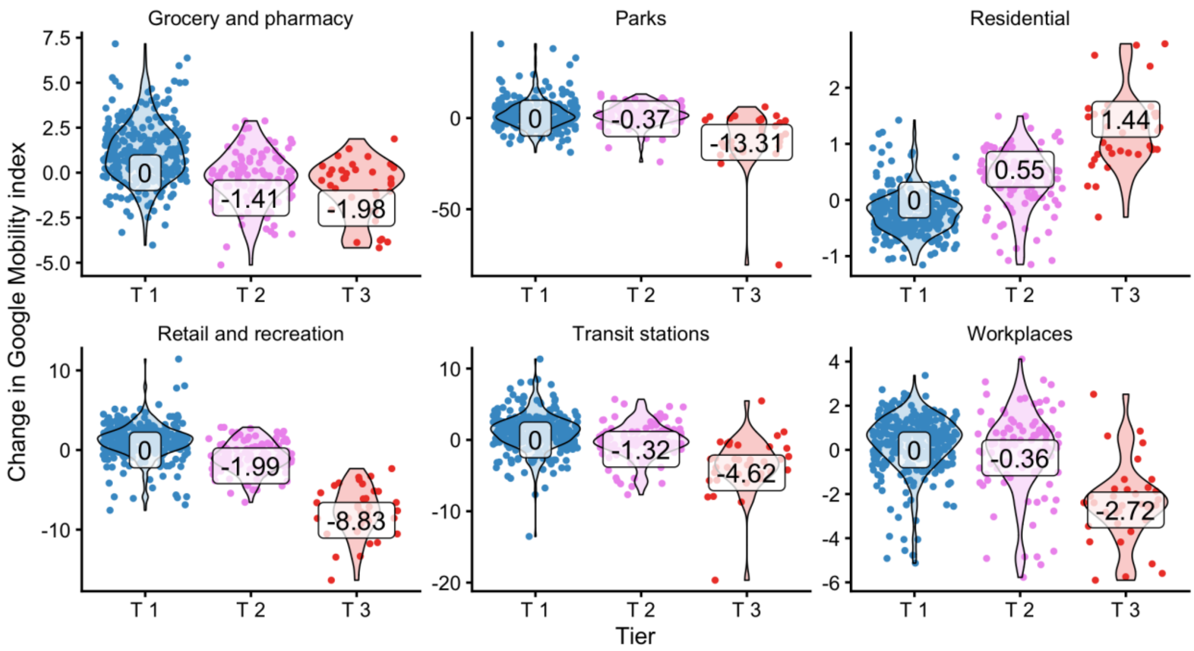

Fig. S2. Impact of tiered restrictions on mobility indices as measured by Google. Points show the individual within-region estimates of the impact of each tier relative to a pre-tiers baseline, with violin plots showing the distribution of points. Labelled values represent the difference between the mean of each respective tier effect and the mean of the tier effect for Tier 1, which we use to estimate an impact on mobility for Tiers 2 and 3 relative to Tier 1. Note that the Parks and Residential mobility indices are not used to inform changes in contact rates in our analysis.

Impact of lockdown restrictions

In order to estimate the potential impact of lockdown restrictions in England, we used Google Community Mobility indices to estimate the impact of lockdown restrictions that were imposed in Northern Ireland from 16 October 2020 and in Wales from 23 October 2020. Additionally, after this analysis was originally conducted, we used the same methodology to estimate the impact of the lockdown as enacted in England. We began by averaging across the mobility indices for each local region in Northern Ireland, Wales, or England, weighting each local region by population, to obtain an overall series of daily mobility indices for each country. Then, we selected a week's worth of data to use as a baseline for comparison for each country's lockdown; the baseline periods were 9–15 October for Northern Ireland, 16–22 October for Wales, and 17–22 October for England. To analyse mobility changes during the lockdown periods, we focused on 17–30 October for the circuit breaker in Northern Ireland, 24 October–6 November for the firebreak in Wales, and 5–25 November for the lockdown in England, subtracting each within-lockdown day's mobility indices from the mobility indices measured for the corresponding weekday during the baseline period. Matching by weekday in this manner allowed us to control for day-of-week effects; we chose a baseline period for England further back in time relative to the lockdown than we did for Northern Ireland and Wales so that the baseline period in England would not overlap with the half-term school break during 24 October – 1 November. The average difference for each indicator and country was assumed to capture the impact of lockdown restrictions (Fig. S3). We found a substantially greater impact of lockdown restrictions in Wales compared to Northern Ireland, with the retrospectively analysed lockdown in England having an intermediate effect between the two.

Imposition of tiered restrictions

Regarding our analysis of tiered restrictions in England, UK government guidelines state that a local authority will be considered for admission to Tier 2 when the incidence of cases (as detected by tests administered by the NHS (Pillar 1) and commercial partners (Pillar 2)) exceeds 100 new cases per 100,000 population over a period of one week. There is no official guidance on the threshold needed to progress to Tier 3, but inspection of the seven-day case rates in regions that were placed under Tier 3 restrictions suggests a threshold of approximately 300 cases per 100,000 population over a seven-day period. Using ONS estimates for viral prevalence, we estimated that approximately 1 in 7 SARS-CoV-2 infections in the UK is detected by Pillar 1 or Pillar 2 surveillance. We fixed model thresholds for progressing into Tiers 2 or 3 at 700 new infections per 100,000 population and 2,100 new infections per 100,000 population over a seven-day period, respectively. In keeping with stated UK government guidelines, we assumed that tier classifications would be reviewed after 28 days for potential downgrading of a tier (i.e. from Tier 3 or Tier 2 to a lower tier). However, we assumed that regions could move into a higher tier immediately if they passed the threshold for a higher tier before the 28-day review period had passed.

When simulating the impact of tiered restrictions and lockdown on transmission dynamics, we use both the mean estimated impact for each mobility index as well as the standard error of each impact. When running simulations, we draw normally-distributed random numbers for each run of the simulation to incorporate this uncertainty, and apply these to the estimated impacts of each type of restriction. For example, if the random number is +1 for Tier 2, then we add one standard error to each mobility indicator whenever a region is under Tier 2

restrictions. We assume that these errors are correlated across mobility indicators within a given restriction, i.e., if the effect of Tier 2 on workplace contacts is overestimated by one standard error, we assume that the effect of Tier 2 on grocery and pharmacy contacts is also overestimated by one standard error, but uncorrelated between Tier 2, Tier 3, and lockdowns.

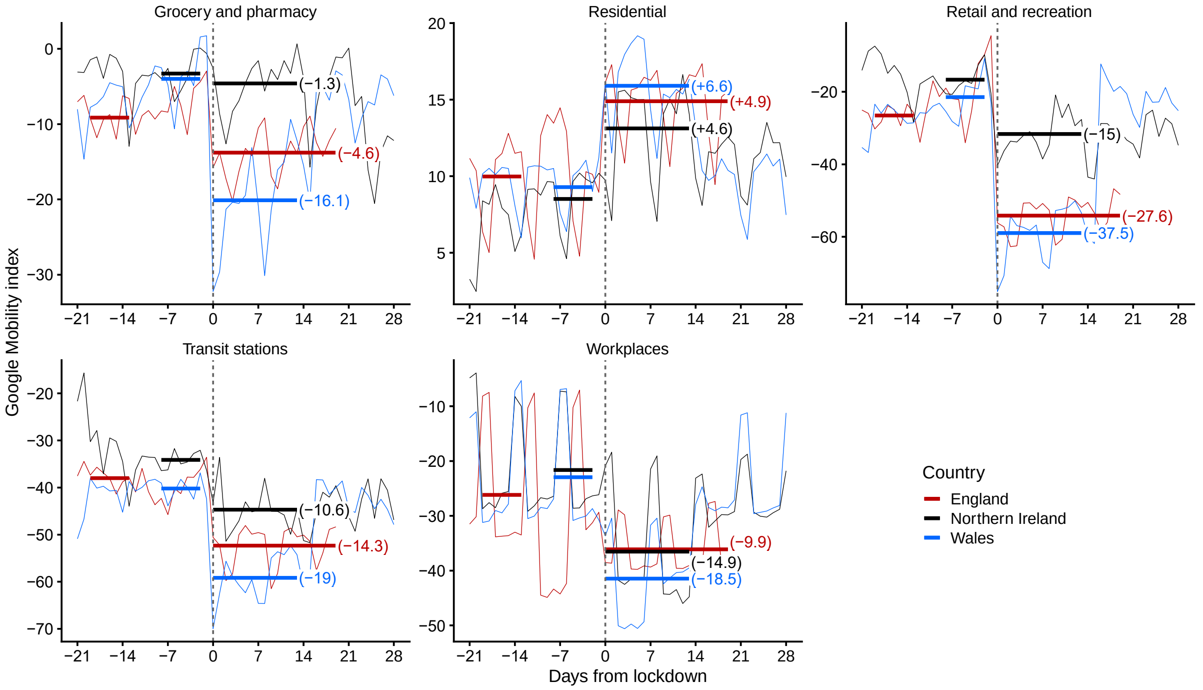

Fig. S3. Estimated mean change in mobility indices following lockdown restrictions imposed in Northern Ireland, Wales, and England, relative to the first full day of lockdown (day 0). Average mobility indices for each country are shown using fine lines, while thick horizontal lines show the average mobility indices for pre-lockdown and lockdown periods. The vertical dashed line marks the first full day of lockdown. Labelled numbers in parentheses show the relative change for each mobility index between pre-lockdown and lockdown. Note that the Residential mobility index is not used to inform changes in contact rates in our analysis.

Sensitivity analyses with waning immunity and seasonal patterns of transmission

As a sensitivity analysis, we estimated how the effect of interventions might vary under a scenario with a seasonal increase in contact rates and/or with waning immunity to reinfection by SARS-CoV-2. In both cases, we assumed that seasonal increases or waning would start only on 1 October 2020 and carry forward for the rest of the simulation. We assumed that seasonal changes in contact rates were uniform across age groups and followed a sinusoidal curve with an amplitude of 10% (i.e. peak-to-trough difference of 20%), peaking on 1 January 2021, and that waning immunity followed an exponential distribution with an average duration of protection of 40 weeks; estimates for seasonality and waning are adopted from a previous study[6].

PCR positivity

We obtained estimates for the probability of testing PCR positive on a given day since infection from an unpublished study which included both symptomatic and asymptomatic individuals. We assumed that the time from infection to PCR positivity and the time from PCR positivity to loss of PCR positivity were uncorrelated, and could each be described by a separate gamma distribution with a mean and shape parameter to be estimated. Adopting uniform priors for the mean and shape of both gamma distributions, we performed Bayesian inference using MCMC to estimate the parameters of both gamma distributions, which yielded an average duration of PCR positivity of 8.5 days.

Model fitting

To fit the model to data on deaths, hospital admissions, hospital bed and ICU bed occupancy, PCR positivity, and seroprevalence for each of the 7 NHS England regions, we performed Bayesian inference using Markov chain Monte Carlo, employing the Differential Evolution MCMC algorithm[7]. For each posterior sample, we simulated epidemics from 1 January to 24 October 2020, using data that were current as of 23 October 2020. We used Google Community Mobility data up to 13 October 2020, the day before the implementation of tiered restrictions in England, to capture how interpersonal contact rates changed over the course of the epidemic, as detailed above; from 14 October 2020, we assumed that mobility indicators were “frozen” at their mean values for each region as measured over the week of 10-16 October, with further changes dictated by the introduction of tiered restrictions and lockdowns, as well as sinusoidally varying seasonal changes in transmission rates for sensitivity analyses with seasonally-varying transmission.

For deaths, hospital admissions, hospital bed occupancy and ICU bed occupancy, we used a negative binomial likelihood with size parameter fixed at 20 for each daily data point. For seroprevalence and PCR prevalence, we used a skew-normal likelihood for each data point fitted to produce the same mean and 95% confidence interval as was reported for the data and took the expected value of the model prediction over the date range during which the prevalence was measured.

As part of model estimation, we separately fit for each region: the start time of community transmission; the basic reproduction number R_0 prior to any changes in mobility or closure of schools; the delay from infection to hospital admission, to ICU admission, and to death; a

region-specific relative probability of hospital admission and of ICU admission given infection; the relative infection fatality ratio at the start and at the end of the simulation period, as fatality due to COVID-19 has dropped substantially over time in the UK; a decreasing rate of effective contact between individuals over time, representing better practices of self-isolation and precautions against infection taken by individuals over the course of the year; coefficients determining the relative mobility of younger people, around age 20, relative to the rest of the population, for the months of July, August, and September onwards; and the magnitude and timing of a boost to R_0 around the end of summer, which we hypothesise is related to the opening of schools, but which was not fully captured in our model by the resumption of school-specific contacts on 1 September. Full details of all fitted parameters, along with prior distributions assumed for each parameter, are in **Table S1**.

We use two parametric functions extensively in parameterising the model. The first,

$$logistic(x) = (exp\, x)/(1 + exp(x))$$

is the standard logistic curve. The second,

$$asc(x, y_0, y_1, s_0, s_1) = y_0 + (y_1 - y_0)(logistic(s_o + x(s_1 - s_0)) - logistic(s_0))/(logistic(s_1) - logistic(s_0))$$

is a logistic-shaped curve parameterised to be a smooth S-shaped function of x from 0 to 1, which goes from y_0 at $x = 0$ to y_1 at $x = 1$, with an inflection point at $x = -s_0/(-s_0 + s_1)$ if $s_0 < 0$ and $s_1 > 0$.

Basic epidemiological parameters were broadly informed from the literature and previously reported. We assumed an average incubation period for SARS-CoV-2 of 5 days[8], an average infectious period of 5 days[9], with roughly half of transmission from symptomatic individuals occurring before symptom onset[10] and an age-specific susceptibility to infection according to a previously-published analysis of case data from 6 countries[11]. Note that while we assume that an individual's infectiousness is constant over the duration of their infectious period, when averaged over the population of all individuals, for which the duration of infectiousness is drawn from a distribution, the peak of infectiousness occurs around the onset of symptoms, as has been suggested by studies of SARS-CoV-2 transmission[10] (see **Assumptions around peak of infectiousness**, below). All parameters that we adopted as assumptions are given in **Table S2**.

Projections for lockdown as enacted in England

At the end of November, we assessed the actual impact of the English lockdown on observed mobility and used these estimates to update our projections (see **Impact of lockdown restrictions**, above). The projected impact of the English lockdown on estimated cumulative deaths over the winter period, hospital pressure and time spent under different restrictions are similar to our base-case analysis, but the lower impact of the lockdown means that the distinction between the different policies (in terms of timing and duration) is less marked (**Figs. S4, S5**).

Table S1. Details of fitted parameters.

Parameter	Description	Prior distribution	Notes
`tS`	Start date of epidemic in days after 1 January 2020	$\sim uniform(0,60)$	Determines date at which seeding begins in region; starting on this date, one random individual per day contracts SARS-CoV-2 for 28 days
`u`	Basic susceptibility to infection	$\sim normal(0.07, 0.01)$	Determines basic reproduction number R_0
`death_mean`	Mean delay in days from start of infectious period to death	$\sim normal(15,2)$	Prior informed by analysis of CO-CIN data
`death_shape`	Shape parameter of gamma distribution for delay from start of infectious period to death	$\sim normal(1.9,0.2)$	Prior informed by analysis of CO-CIN data
`admission`	Mean delay in days from start of infectious period to hospital admission	$\sim normal(7.5, 1)$	Delay is assumed to follow a gamma distribution with shape parameter 0.71. Prior and shape of distribution informed by analysis of CO-CIN data.
`icu_admission`	Mean delay in days from start of infectious period to ICU admission	$\sim normal(11.1, 1)$	Delay is assumed to follow a gamma distribution with shape parameter 1.91. Prior and shape of distribution informed by analysis of CO-CIN data.
`hosp_rlo`	Log-odds of hospital admission, relative to age-specific probabilities of hospital admission given infection derived from Salje et al. [REF].	$\sim normal(0, 0.1)$	Based on Salje et al.[12], we assumed that the basic shape of the age-specific probability of hospitalisation given infection was $logistic(-7.37 + 0.068a)$, where *a* is the individual's age in years. This overall relationship is then adjusted according to the `hosp_rlo` parameter.
`icu_rlo`	Log-odds of ICU admission, relative to age-specific probabilities of ICU admission given hospital admission derived from CO-CIN data.	$\sim normal(0, 0.1)$	We fit a spline to CO-CIN data on hospital admission and ICU admission by age to derive the basic age-specific probability of ICU admission, which was then adjusted based on the `icu_rlo` parameter.
`cfr_rel`	Relative fatality rate of COVID-19 at beginning of 2020	$\sim normal(1, 0.05)$	Based on Levin et al.[13], we assumed the basic shape of the age-specific infection fatality ratio of SARS-CoV-2 was $logistic(-7.56 + 0.121a)$ (see entry for `hosp_rlo`). This is multiplied by `cfr_rel` to adjust the fatality rate for each region.
`cfr_rel2`	Relative fatality rate of COVID-19 at end of 2020	$\sim normal(0.45, 0.01)$	Based on CO-CIN data[14], we estimated that the mortality rate of COVID-19 decreased by approximately 55% by September 2020 relative to the beginning of the year. The product of `cfr_rel` and `cfr_rel2` gives the mortality rate by September. Specifically, the IFR is multiplied by a factor $asc(t/366, cfr\_rel, cfr\_rel \times cfr\_rel2, -2.9, 7.8)$ where *t* is the time in days since 1 January 2020.
`contact_final`	Relative rate of effective contact at end of 2020	$\sim normal(1, 0.1) \leq 1$	To capture continued low incidence of SARS-CoV-2 infection in spite of rising contact rates as shown by mobility data and social contact surveys, we assume that the effective contact rate over time is multiplied by a factor
`contact_s0`	Parameter for curve specified by `contact_final`	$\sim exponential(0.1)$	

contact_s1	Parameter for curve specified by contact_final	$\sim exponential(0.1)$	$asc(t/366, 1, contact_{final}, -contact_{s0}, contact_{s1})$, where *t* is time in days since 1 January 2020.
concentration1	Increased contact among young people in July	$\sim normal(2, 0.5) \geq 2$	Because initial increases in SARS-CoV-2 prevalence from July in England were especially apparent in young people, we allow increases in mobility to be more emphasized in young people starting from July. We model a relative contact-rate multiplier for individuals of age *a* as $beta(a/100 \vert \alpha = 0.2(k-2)+1, \beta = 0.8(k-2)+1)$, where *k* is the concentration parameter and *beta* is the beta distribution probability density function. This gives flat contact rates across age groups when *k* = 2, and relatively higher contact rates in individuals around age 20 when *k* > 2.
concentration2	Increased contact among young people in August	$\sim normal(2, 0.4) \geq 2$	
concentration3	Increased contact among young people from September	$\sim normal(2, 0.2) \geq 2$	
sep_boost	Increase in transmission around 1 September 2020	$\sim normal(1, 0.05)$	After the date specified by sep_when, transmission is multiplied by the factor sep_boost. This is to capture a sudden increase in transmission rates observed around 1 September in England.
sep_when	Date of increase in transmission	$\sim uniform(224, 264)$ (i.e. 12 Aug–21 Sep)	

Table S2. Model parameters not subject to fitting.

Parameter	Description	Value	Notes
d_E	Latent period (E to I_P and E to I_S; days)	$\sim gamma(\mu = 2.5, k = 4)$	Set to 2.5 so that incubation period (latent period plus period of preclinical infectiousness) is 5 days [8]
d_P	Duration of preclinical infectiousness (I_P to I_C; days)	$\sim gamma(\mu = 2.5, k = 4)$	Assumed to be half the duration of total infectiousness in clinically-infected individuals [10]
d_C	Duration of clinical infectiousness (I_C to R; days)	$\sim gamma(\mu = 2.5, k = 4)$	Infectious period set to 5 days, to result in a serial interval of approximately 6 days[15–17]
d_S	Duration of subclinical infectiousness (I_S to R; days)	$\sim gamma(\mu = 5.0, k = 4)$	Assumed to be the same duration as total infectious period for clinical cases, including preclinical transmission
y_i	Probability of clinical symptoms given infection for age group *i*	Estimated from case distributions across 6 countries	[11]
f	Relative infectiousness of subclinical cases	50%	Assumed[9,11]
c_{ij}	Number of age-*j* individuals contacted by an age-*i* individual per day, prior to changes in mobility	UK-specific contact matrix	[2]
N_i	Number of age-*i* individuals	From demographic data	[18]
Δt	Time step for discrete-time simulation	0.25 days	
$P(ICU)_i$	Proportion of hospitalised cases that require critical care for age group *i*	Estimated from CO-CIN data	[14]
w_s	Waning rate of seropositivity	224 days^{-1}	Estimated from serology data
los_{hosp}	Length of stay in hospital	$\sim lognormal(\mu_{log} = 11.08, \sigma_{log} = 1.20)$	Estimated from CO-CIN data[14]
los_{icu}	Length of stay in ICU	$\sim lognormal(\mu_{log} = 13.33, \sigma_{log} = 1.25)$	Estimated from CO-CIN data[14]
$detect_0$, $detect_1$, $detect_{s0}$, $detect_{s1}$	Delay from hospital admission to SARS-CoV-2 test	$detect_0 = 14$<br>$detect_1 = 1$<br>$detect_{s0} = 5.86$<br>$detect_{s1} = 33.4$	To capture substantial delays in testing at the beginning of the epidemic in the UK, we assume that the delay from hospital admission to confirmed SARS-CoV-2 infection is $asc(t/366, detect_0, detect_1, -detect_{s0}, detect_{s1})$, where *t* is time in days since 1 January 2020. Estimated from a previous round of model fitting.

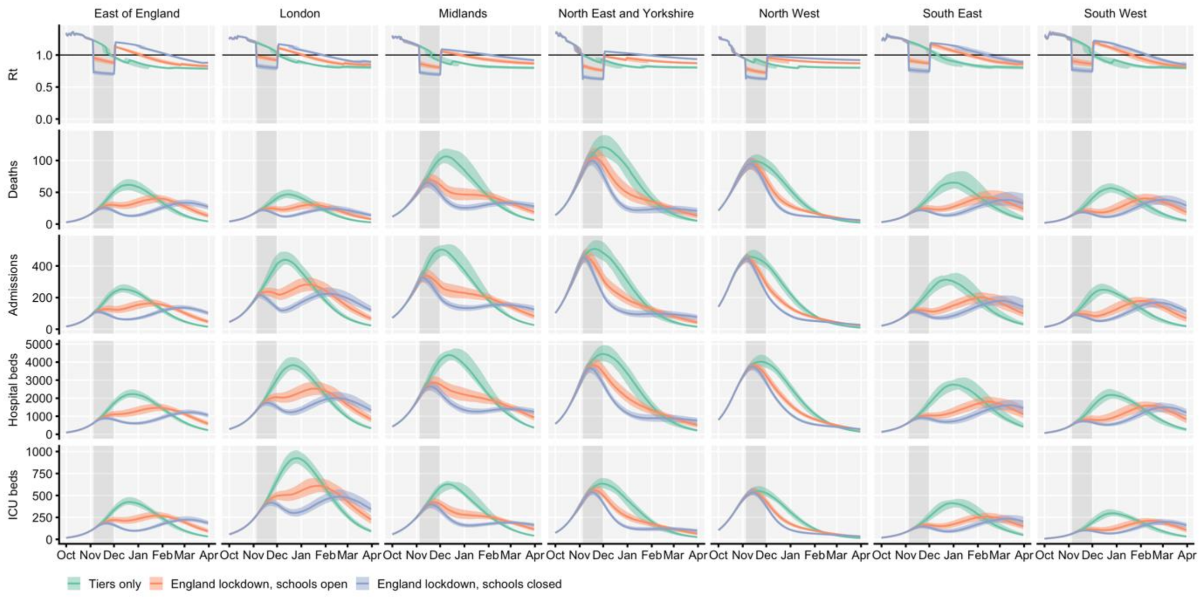

Fig. S4. Projected impact of the enacted lockdown in England. The effective reproduction number Rt, as well as the daily incidence of deaths and hospital admissions and the daily prevalence of occupied hospital and ICU beds is contrasted across seven NHS regions for three different scenarios: (i) tiered restrictions only, (ii) lockdown with schools open, (iii) lockdown with schools closed. Lockdowns extend from 5 November to 2 December 2020 inclusive. Lines and shaded ribbons give the median and 95% credible interval for plotted quantities, while the shaded background area shows the lockdown period. Step changes in Rt show the introduction or relaxation of tiered restrictions and lockdown measures.

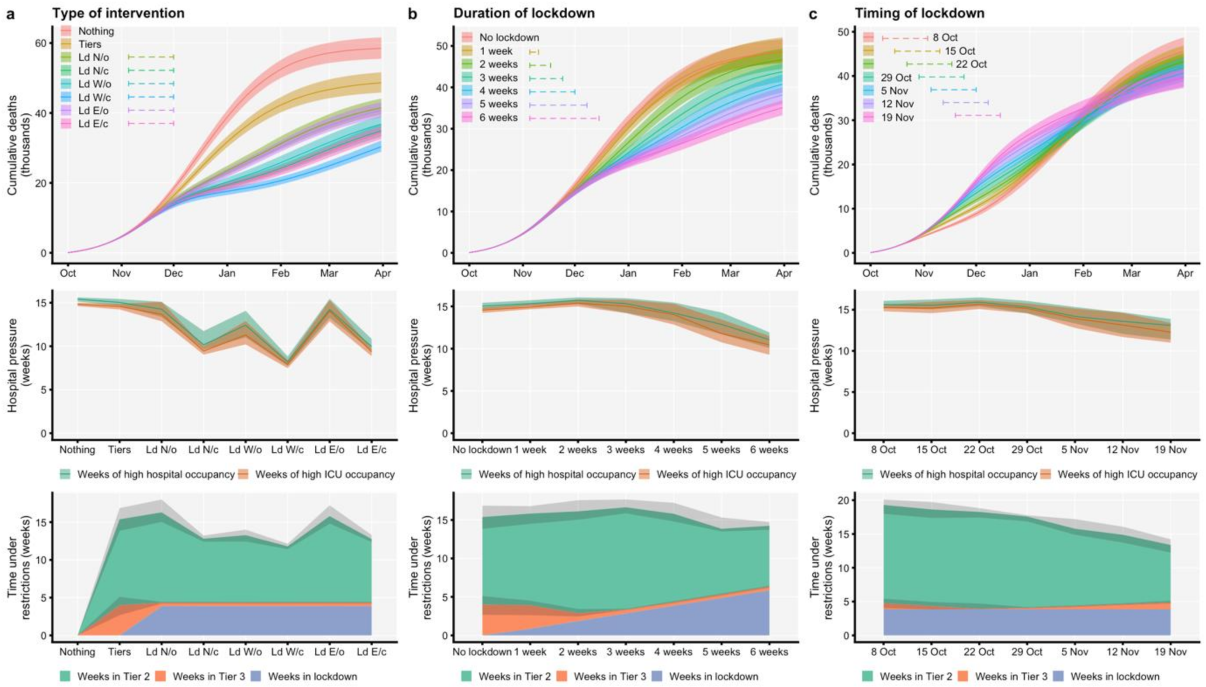

Fig. S5. Contrasting alternative intervention strategies for the lockdown as enacted in England. **(a)** Type of intervention: baseline epidemic ("Baseline"), tiered restrictions only ("Tiers"), or lockdowns (Ld N/o, etc.; see Fig 3, main text). **(b)** Duration of lockdown: with tiered restrictions in place, commencing on 5 November 2020, lockdowns with durations from 0 weeks (No lockdown) to 6 weeks are contrasted. **(c)** Timing of lockdown: four-week lockdowns starting up to four weeks before or two weeks after 5 November 2020 are contrasted. See Fig. 3, main text.

Validation of model predictions

To examine our model's explanatory ability, we assessed our model predictions for when regions of England should pass into higher tiered restrictions using a governmental database of local non-pharmaceutical interventions (**Fig. S6**) and compared our model predictions for mobility changes resulting from the imposition of interventions to Google Mobility indices (**Fig. S7**).

To examine our model's predictive accuracy, we used our estimates for the impact of the second lockdown in England on mobility indices to project our fitted model forward to 7 December 2020, comparing the model predictions to updated data up to this date (**Fig. S8**). This projection uses the same model fit as our main analysis, fitting to data up to 13 October 2020, and from 14 October onwards allows our estimated effects of tiered restrictions and of a four-week lockdown starting on 5 November 2020 to determine model behaviour. The model overestimates ICU bed occupancy during the months of October and November 2020, which may correspond to a sharp decrease in the proportion of hospitalised patients admitted to ICU in these months, according to an analysis of CO-CIN data (**Fig. S9**).

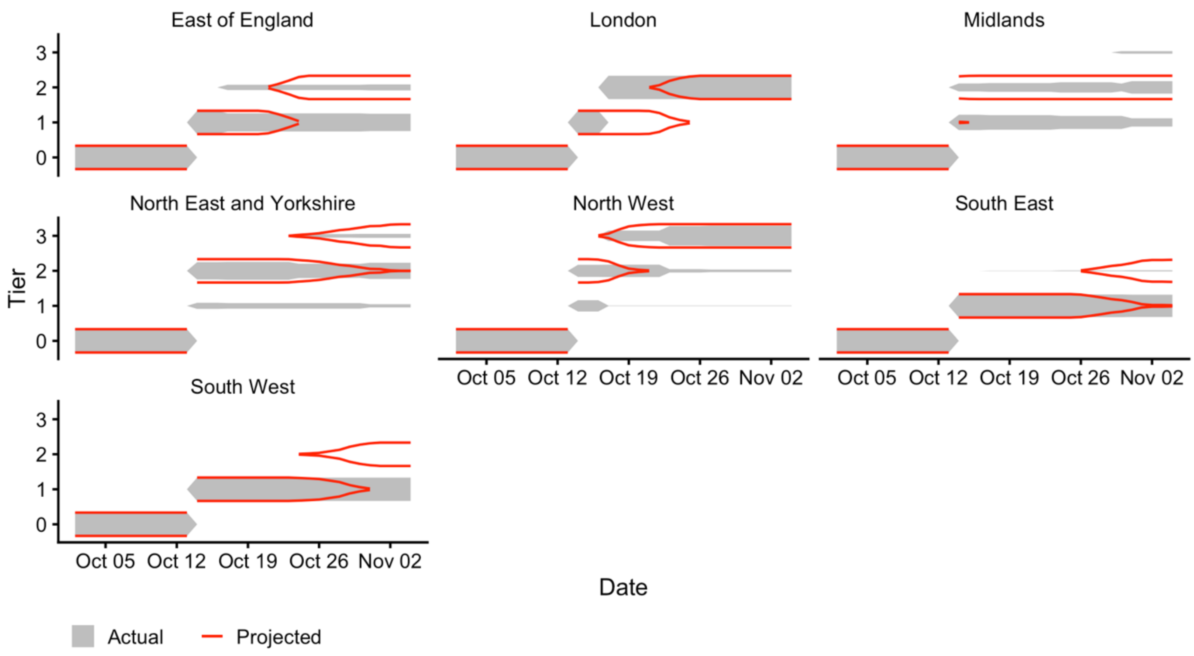

Fig. S6. Comparison of model-predicted versus actual tiered restrictions. The thickness of the grey bars shows the proportion of each NHS region's population under a given tier for each date from 1 October – 4 November 2020, i.e. in the month leading up to the lockdown. The area outlined by the red lines shows the proportion of simulations in which each NHS regions was in a given tier.

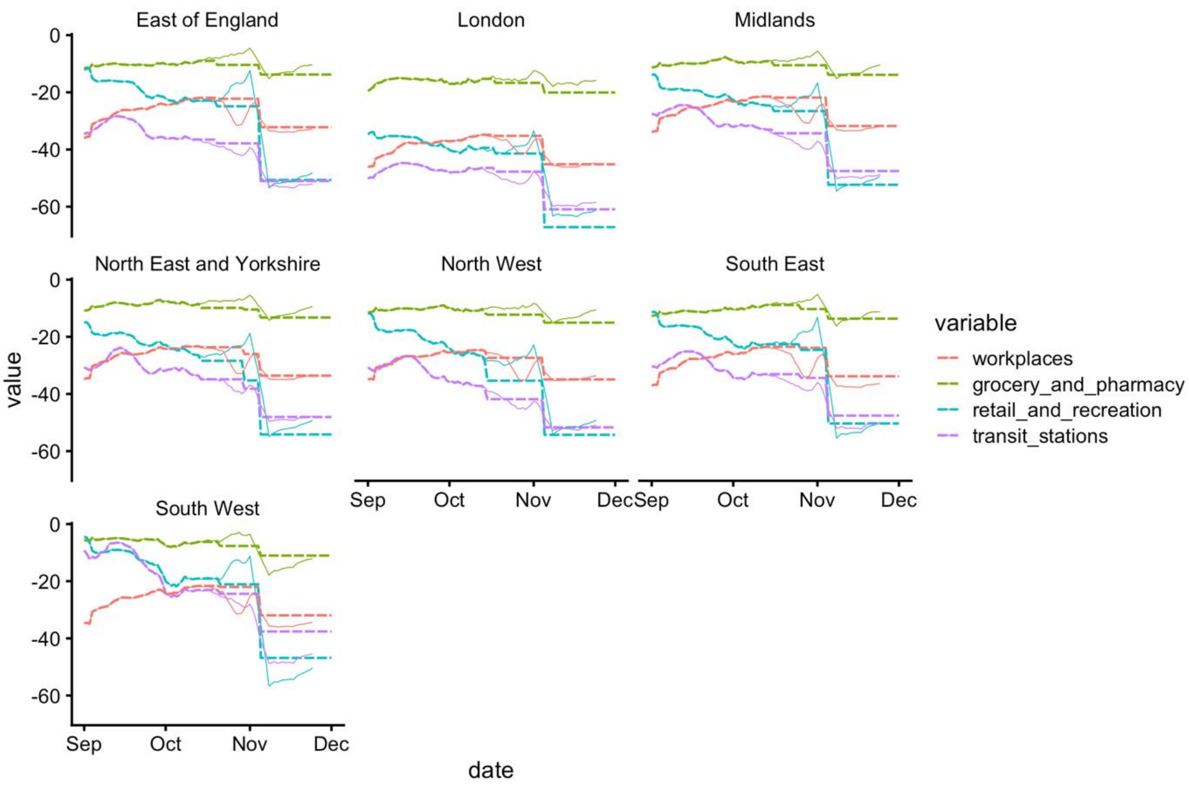

Fig. S7. Comparison of model-predicted versus actual mobility indices. The dashed lines show model predicted mobility indices for each NHS region across four classes of mobility index, using our estimates for the effect of lockdown in England, while the thin solid lines show actual data from Google Mobility for each NHS region. Of note are the substantial changes in movement in the week prior to lockdown, which can partly be accounted for by a half-term break from school during this week. Our model accounts for there being no school contacts during school breaks, but does not account for any decreases in workplace and transit station mobility, or increases in grocery and pharmacy and retail and recreation mobility, which are often associated with school breaks.

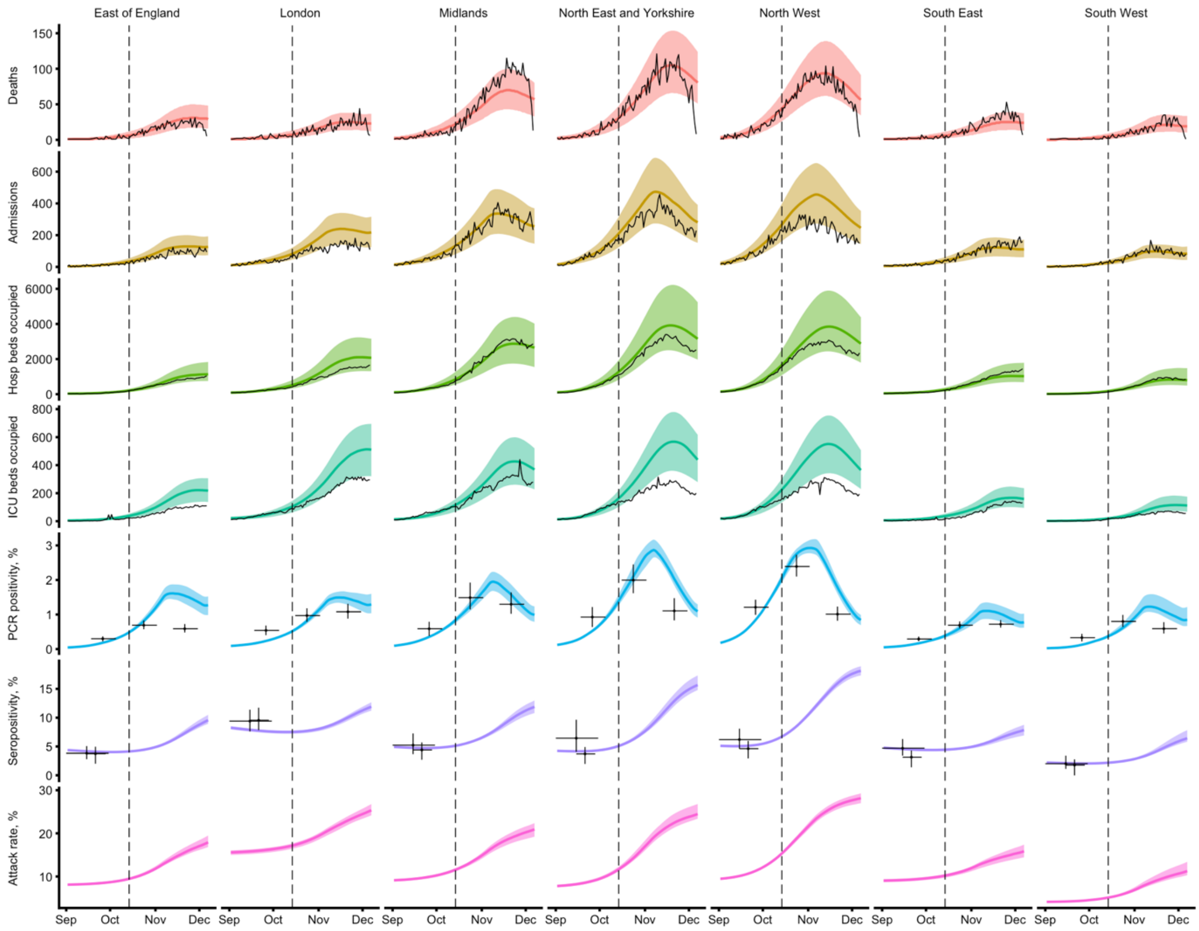

Fig. S8. Comparison of model-predicted versus actual health burdens, PCR positivity, and seropositivity following tiered restrictions and lockdown in England. The dashed lines show the last day of mobility data used by our model; everything forward of this line is a forecast based on the imposition of tiered restrictions on 14 October 2020 and the imposition of a national lockdown on 5 November 2020. Model forecasts are shown to 7 December 2020. Note that deaths data are updated retrospectively since deaths can be reported up to a week later than the date the death occurred, so the last data points for deaths shown here are underestimates.

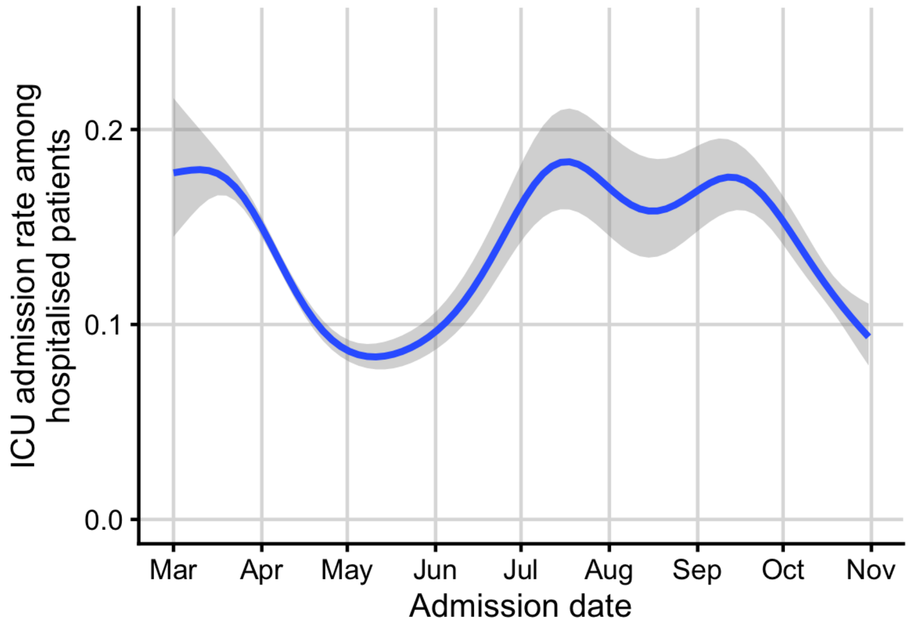

Fig. S9. Changes over time in the proportion of patients hospitalised with COVID-19 who are admitted to ICU, from CO-CIN data (United Kingdom). Our overestimation of ICU occupancy (Fig. S8) is partly explained by a substantial decrease in the ICU admission rate over October, which our model could not have predicted.

Assumptions around peak of infectiousness

There is evidence that the peak of infectiousness in symptomatic individuals occurs around symptom onset[10]. Although we phrase our model assumptions in terms of a constant infectiousness over each individual's infectious period, our model can be interpreted as assuming a peak of infectiousness around the onset of symptoms. That is because while we assume that the average durations of pre-symptomatic and symptomatic infectiousness are each 2.5 days, our model allows for variation among individuals in the duration of these two periods. So, while the infectiousness of any one individual is constant over the duration of their infectious period, when infectiousness is averaged over the population of infectious individuals—who each have different durations of infectiousness—infectiousness is bell-shaped with a peak around the time of symptom onset.

A recent preprint by Ferretti et al.[10] estimates the distribution of infectiousness relative to the time of symptom onset. We compared their distribution of infectiousness to the distribution of infectiousness in our model, according to the assumptions outlined in the previous paragraph, and find that our assumptions around the timing and duration of peak infectiousness are consistent with theirs (**Fig. S10**).

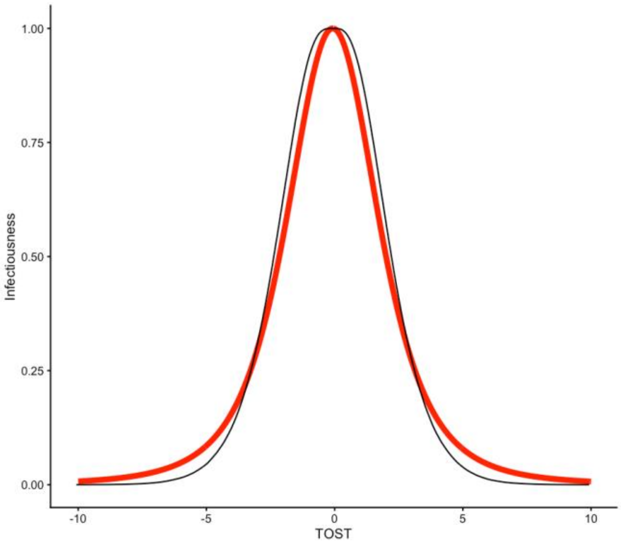

Fig. S10. Peak of infectiousness in our model when averaged across the population (black line) compared to the distribution of infectiousness found by Ferretti et al.[10] relative to the time of symptom onset (TOST, time from onset of symptoms to transmission = 0).

Supplementary figures and tables

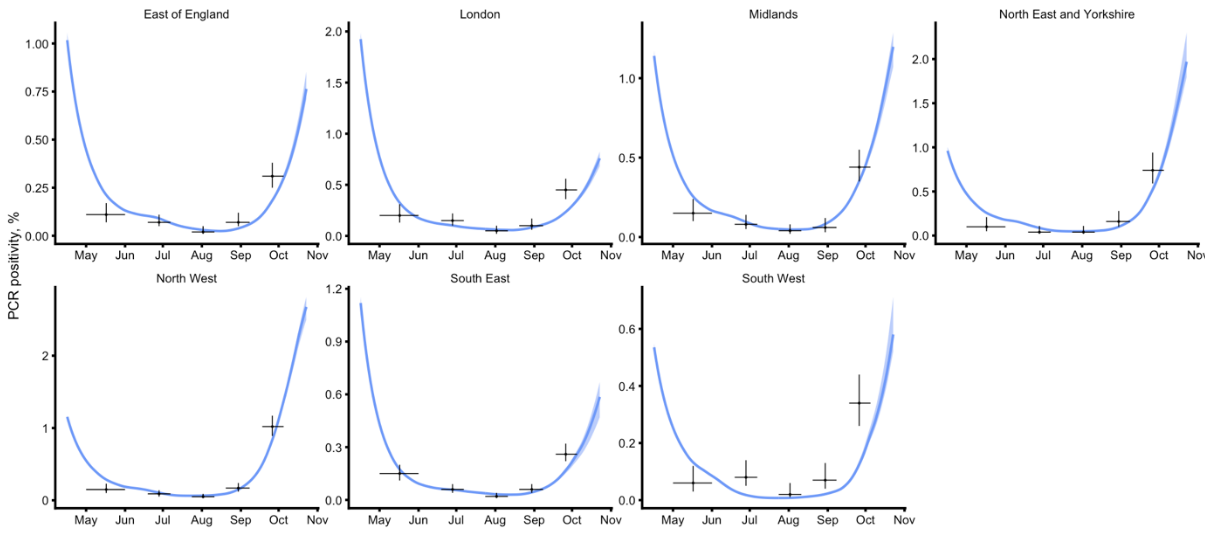

Fig. S11. PCR positivity from model fitting. This is an enlarged version of PCR positivity from Fig. 1, main text. Vertical bars represent 95% confidence intervals for data, horizontal bars represent span of dates measured.

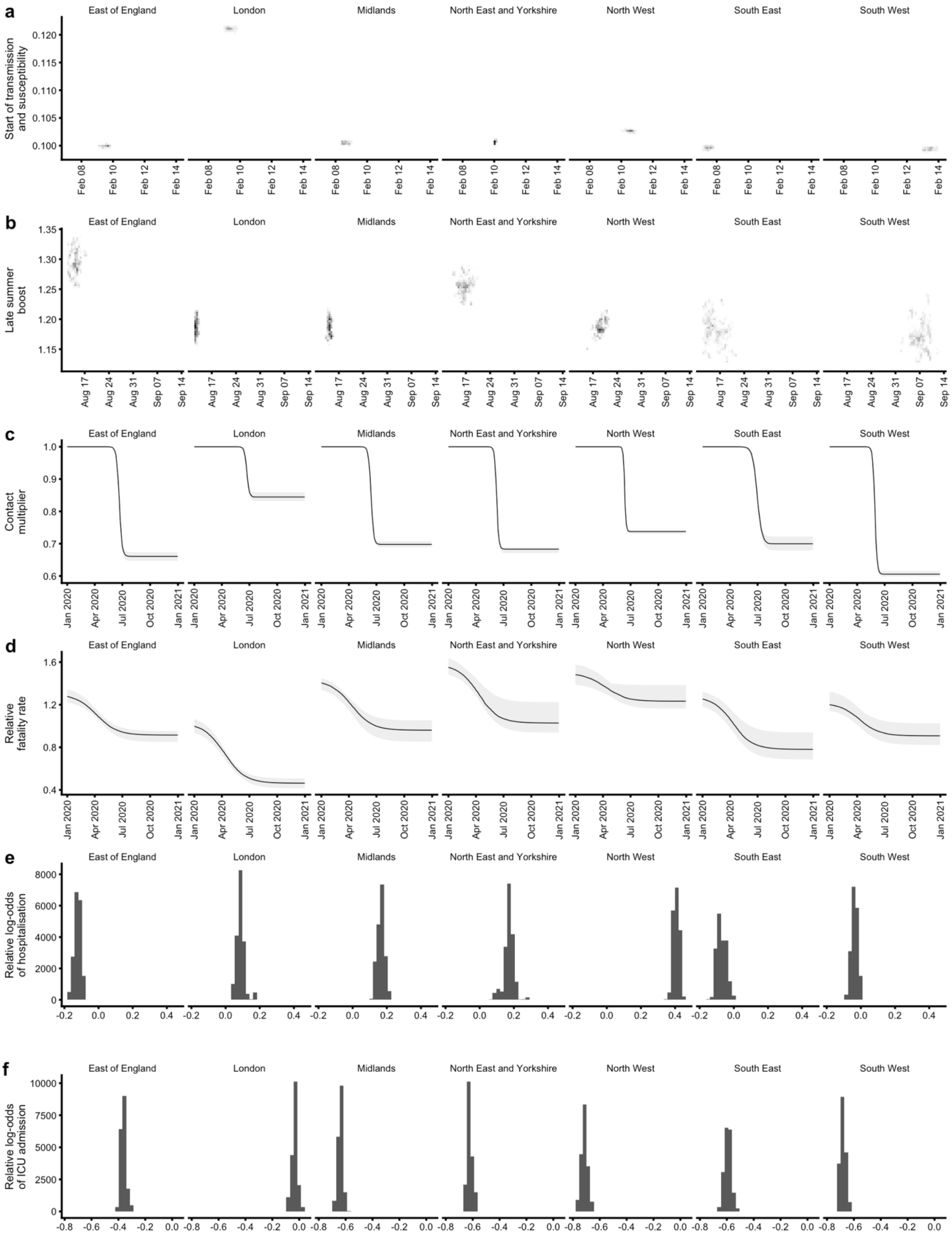

Fig. S12. Posterior distributions from model fitting, pt. 1. See Table 2 for parameter definitions: **(a)** `t_S` (x axis) and `u` (y axis); **(b)** `sep_when` (x axis) and `sep_boost` (y axis); **(c)** `contact_final`, `contact_s0`, `contact_s1`; **(d)** `cfr_rel`, `cfr_rel2`; **(e)** `hosp_rlo`; **(f)** `icu_rlo`.

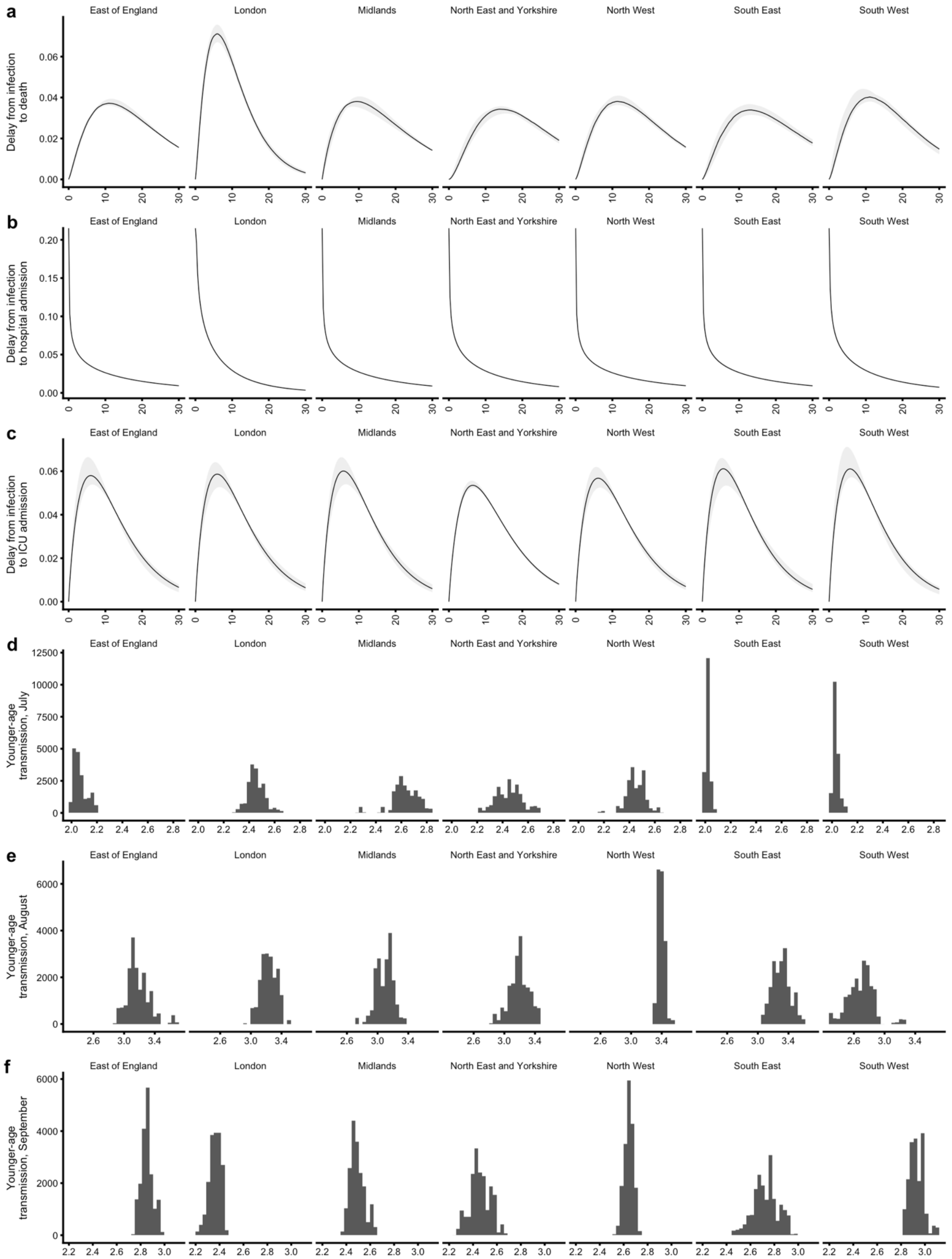

Fig. S13. Posterior distributions from model fitting, pt. 2. See Table 2 for parameter definitions: **(a)** `death_mean`, `death_shape`; **(b)** `admission`; **(c)** `icu_admission`; **(d)** `concentration1`; **(e)** `concentration2`; **(f)** `concentration3`.

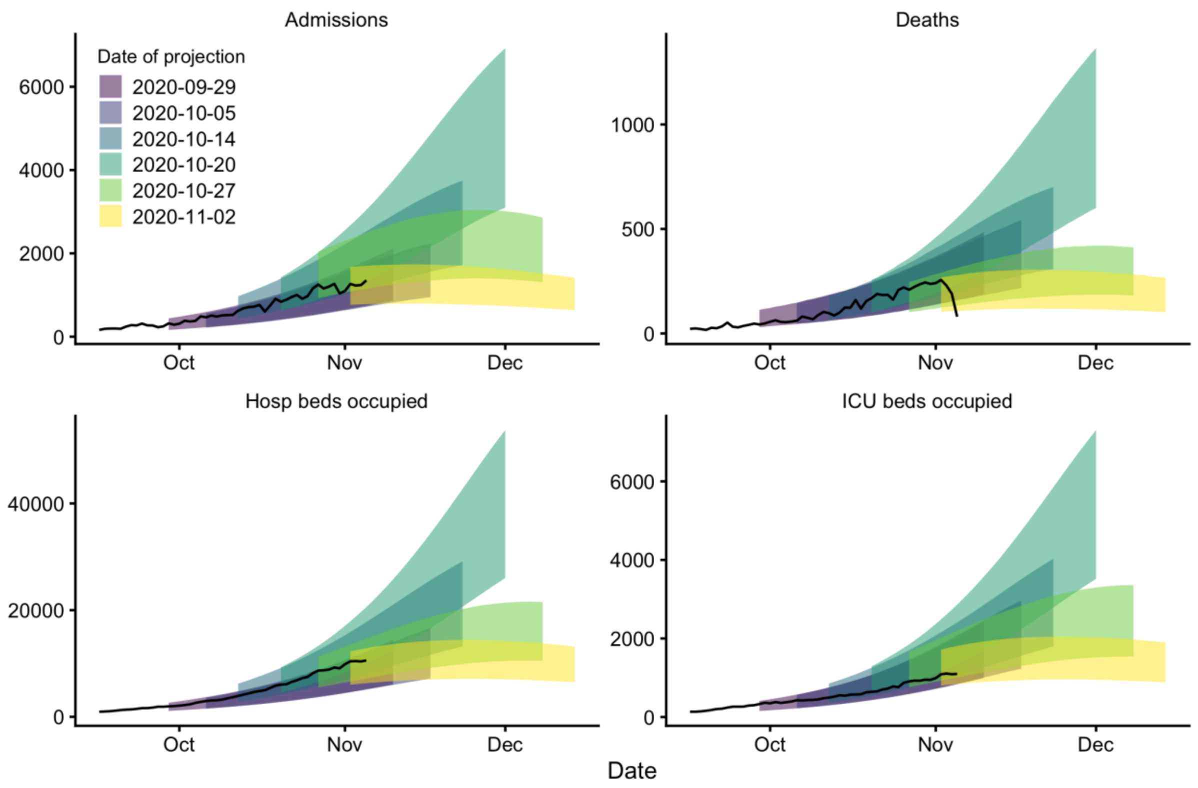

Fig. S14. Model fits to data up to specific dates. These were six-week medium-term projections prepared for SPI-M[19] using the model described in this paper and fitted to data up to the date shown in the legend. Shaded areas show 95% projection intervals. Note that there have been small changes in methodology over time as the model is continually under development. The black lines are the data.

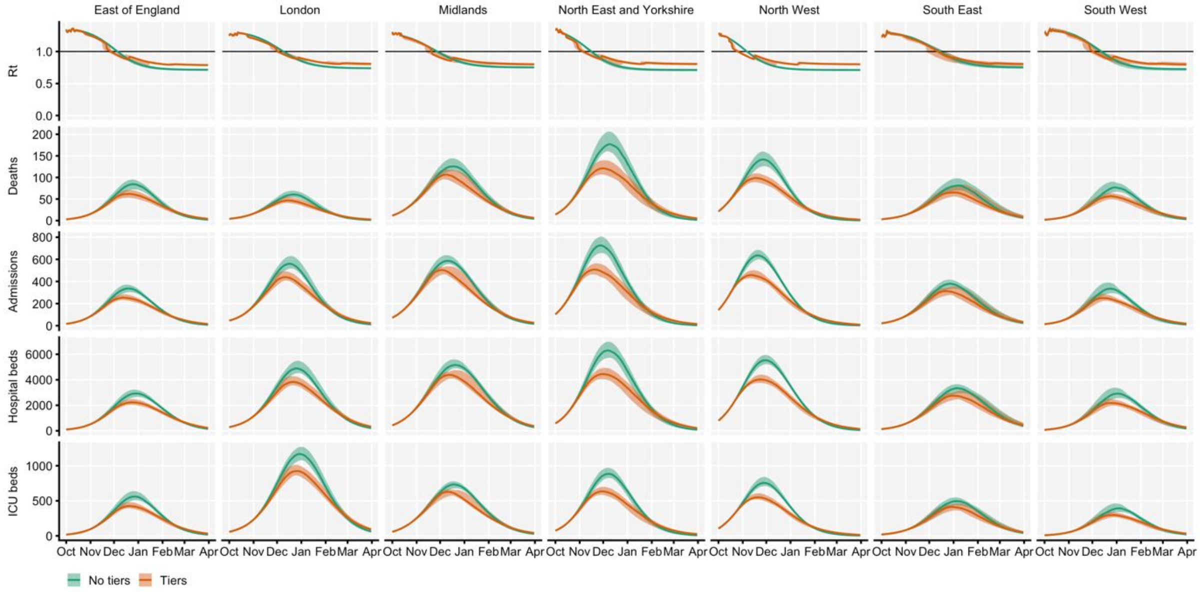

Fig. S15. No tiered restrictions versus tiered restrictions introduced on 14 October 2020. The effective reproduction number Rt, as well as the daily incidence of deaths and hospital admissions and the daily prevalence of occupied hospital and ICU beds is contrasted across seven NHS regions. Lines and shaded ribbons give the median and 95% credible interval for plotted quantities. Step changes in Rt show the introduction or relaxation of tiered restrictions.

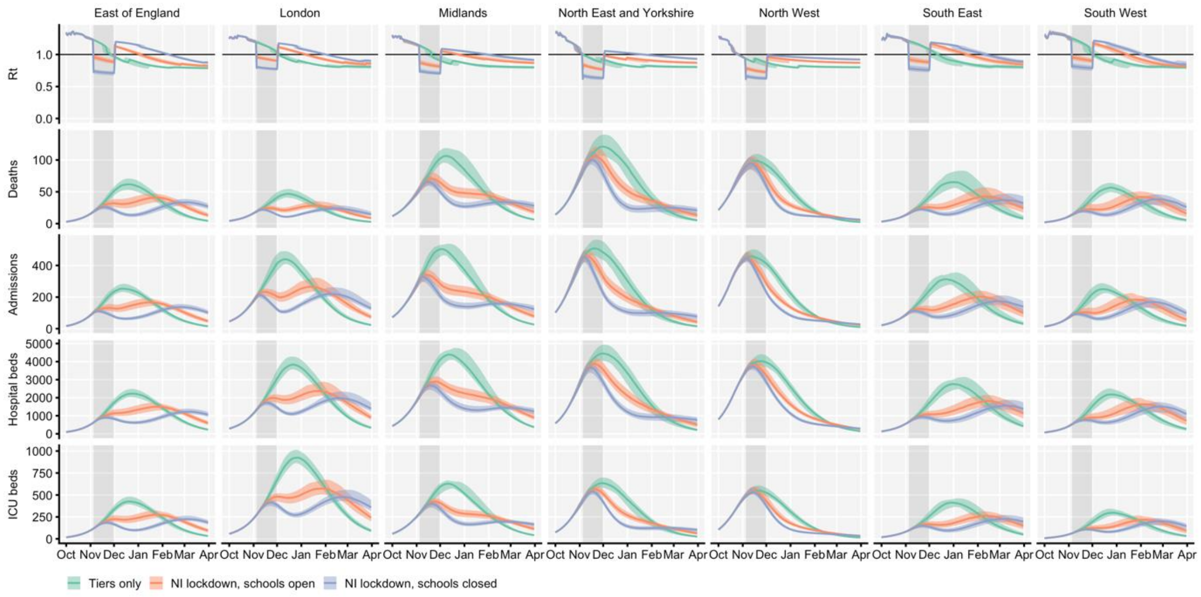

Fig. S16. Projected impact of a Northern Ireland-type lockdown, with and without schools open. The effective reproduction number Rt, as well as the daily incidence of deaths and hospital admissions and the daily prevalence of occupied hospital and ICU beds is contrasted across seven NHS regions. Lockdowns extend from 5 November to 2 December 2020. Lines and shaded ribbons give the median and 95% credible interval for plotted quantities, while the shaded background area shows the lockdown period. Step changes in Rt show the introduction or relaxation of tiered restrictions and lockdown measures.

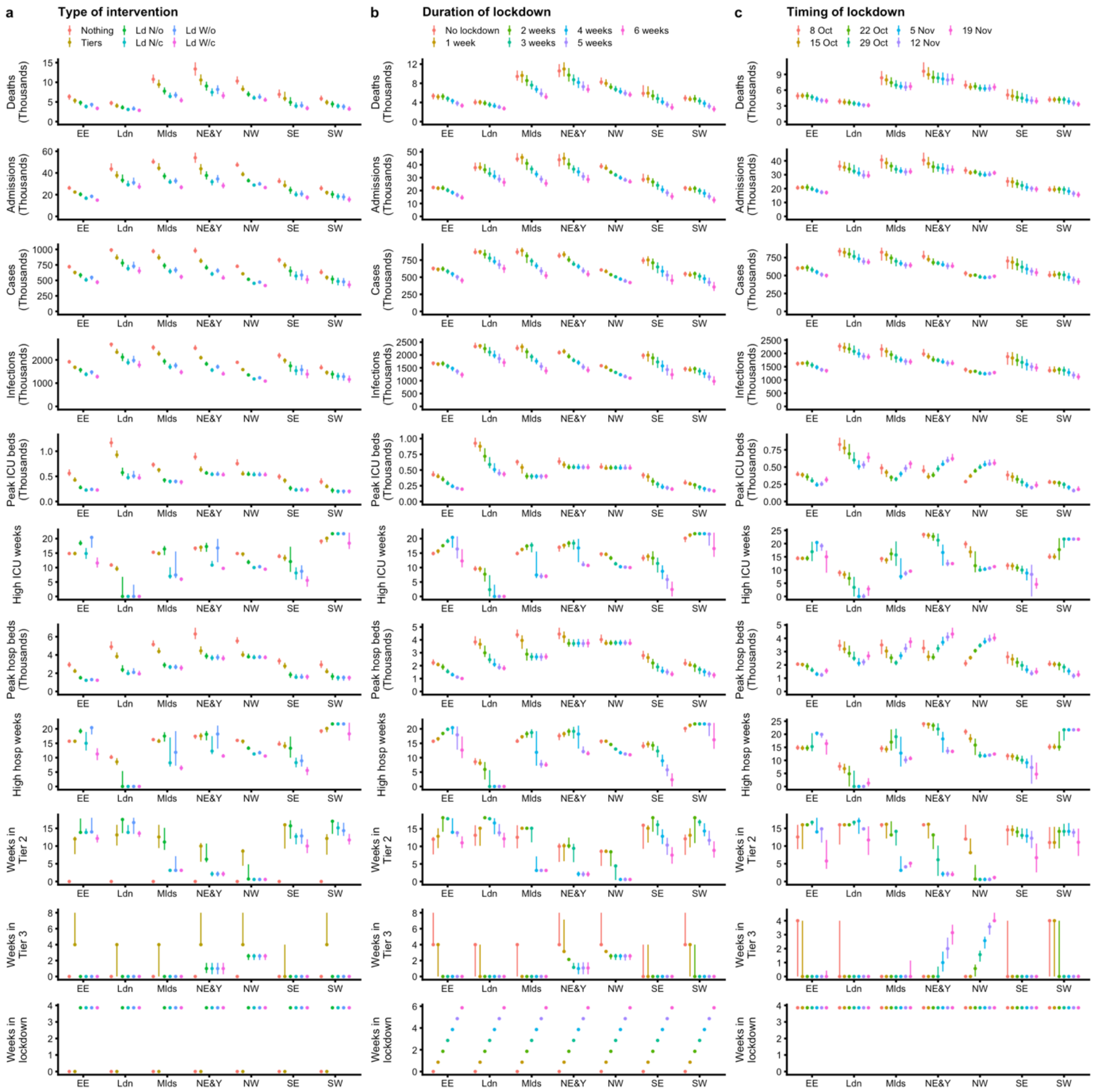

Fig. S17. Region-specific alternative scenarios. These show region-specific values for the scenarios compared in Fig. 3 of the main text. Points and line ranges show median and 95% projection intervals.

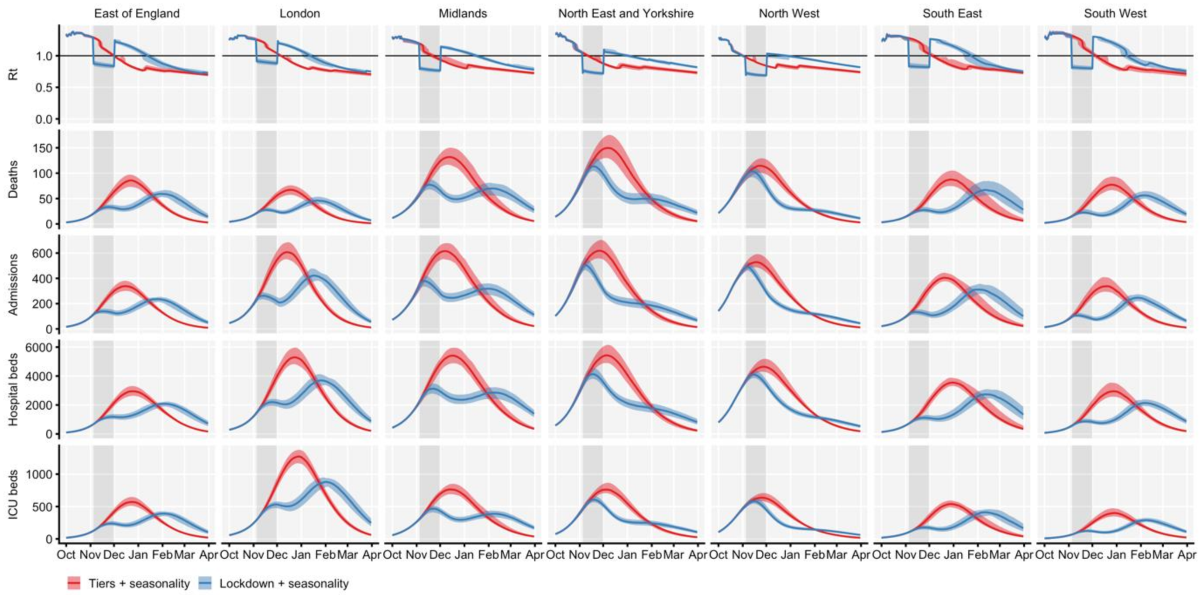

Fig. S18. Alternative scenario: tiered restrictions only versus lockdown, with seasonality. The effective reproduction number Rt, as well as the daily incidence of deaths and hospital admissions and the daily prevalence of occupied hospital and ICU beds is contrasted across seven NHS regions. Lockdowns extend from 5 November to 2 December 2020. Lines and shaded ribbons give the median and 95% credible interval for plotted quantities, while the shaded background area shows the lockdown period. Step changes in Rt show the introduction or relaxation of tiered restrictions and lockdown measures.

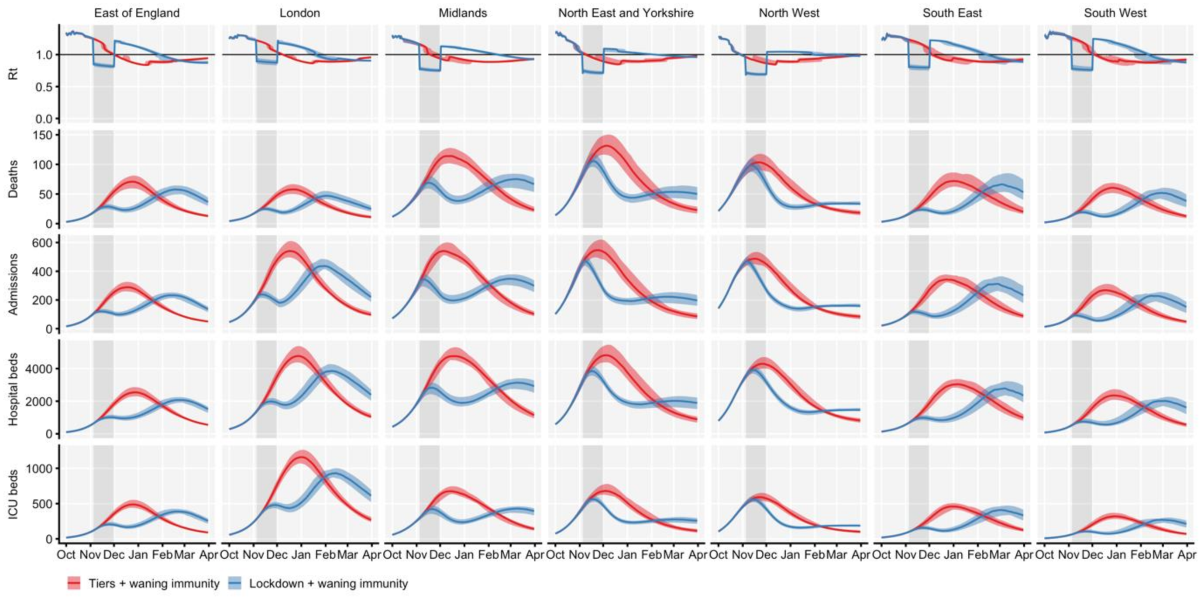

Fig. S19. Alternative scenario: tiered restrictions only versus lockdown, with waning immunity. The effective reproduction number Rt, as well as the daily incidence of deaths and hospital admissions and the daily prevalence of occupied hospital and ICU beds is contrasted across seven NHS regions. Lockdowns extend from 5 November to 2 December 2020. Lines and shaded ribbons give the median and 95% credible interval for plotted quantities, while the shaded background area shows the lockdown period. Step changes in Rt show the introduction or relaxation of tiered restrictions and lockdown measures.

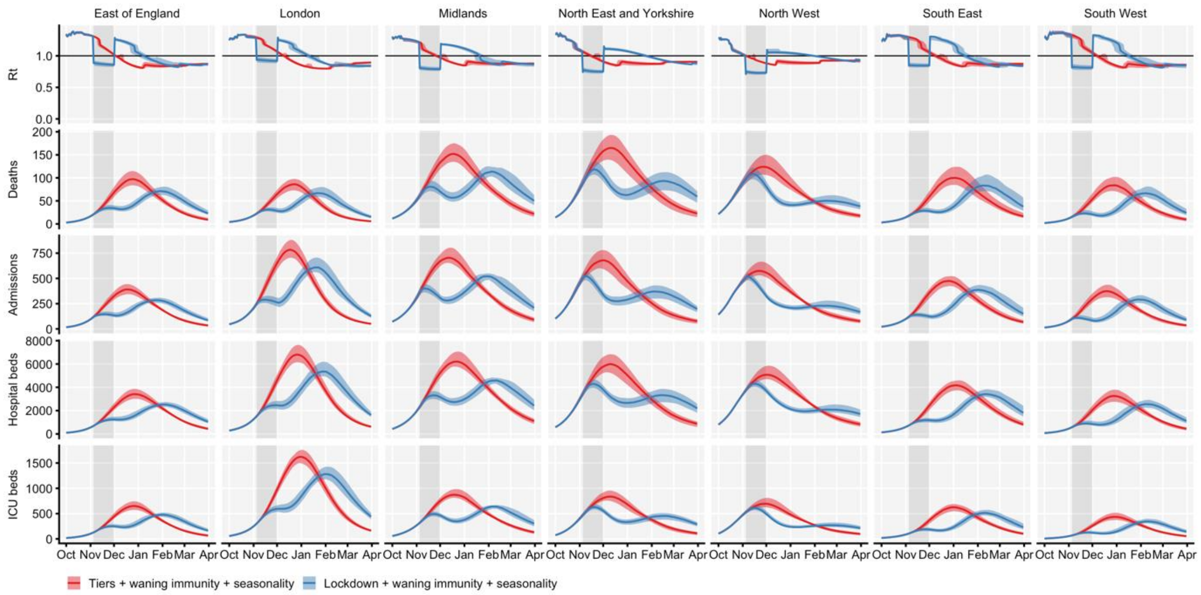

Fig. S20. Alternative scenario: tiered restrictions only versus lockdown, with seasonality and waning immunity. The effective reproduction number Rt, as well as the daily incidence of deaths and hospital admissions and the daily prevalence of occupied hospital and ICU beds is contrasted across seven NHS regions. Lockdowns extend from 5 November to 2 December 2020. Lines and shaded ribbons give the median and 95% credible interval for plotted quantities, while the shaded background area shows the lockdown period. Step changes in Rt show the introduction or relaxation of tiered restrictions and lockdown measures.

Table S3. Region-specific results, baseline scenario (no tiered restrictions, no lockdown). Burdens are summed over the period from 1 Oct 2020 to 31 March 2021. Weeks of high ICU occupancy is calculated by measuring the number of weeks in each region where ICU occupancy is 50% or greater than the peak occupancy during the first wave. Medians and 95% projection intervals shown.

Indicator	England	East of England	London	Midlands	North East and Yorkshire	North West	South East	South West
Admissions	280,000 (274,000 - 287,000)	26,200 (24,600 - 28,000)	43,700 (41,900 - 46,500)	50,300 (48,700 - 52,600)	54,000 (49,600 - 58,000)	47,400 (45,900 - 49,800)	32,400 (31,000 - 34,800)	25,800 (24,000 - 27,700)
Deaths	58,500 (55,800 - 61,100)	6,360 (5,590 - 6,930)	4,730 (4,190 - 5,280)	10,800 (9,840 - 11,700)	13,400 (11,900 - 15,000)	10,300 (9,530 - 11,300)	6,910 (6,100 - 8,110)	5,840 (5,320 - 6,580)
Peak ICU (rel. W1)	168% (162 - 174%)	207% (190 - 233%)	101% (94 - 108%)	168% (156 - 176%)	287% (269 - 312%)	212% (202 - 231%)	140% (126 - 154%)	403% (360 - 452%)
Peak ICU requirement	5,000 (4,840 - 5,170)	561 (515 - 631)	1,170 (1,080 - 1,240)	733 (681 - 769)	886 (832 - 966)	755 (720 - 822)	495 (445 - 544)	393 (351 - 442)
Weeks in Tier 2	0 (0 - 0)	0 (0 - 0)	0 (0 - 0)	0 (0 - 0)	0 (0 - 0)	0 (0 - 0)	0 (0 - 0)	0 (0 - 0)
Weeks in Tier 3	0 (0 - 0)	0 (0 - 0)	0 (0 - 0)	0 (0 - 0)	0 (0 - 0)	0 (0 - 0)	0 (0 - 0)	0 (0 - 0)
Weeks in lockdown	0 (0 - 0)	0 (0 - 0)	0 (0 - 0)	0 (0 - 0)	0 (0 - 0)	0 (0 - 0)	0 (0 - 0)	0 (0 - 0)
Weeks of high ICU occupancy	14.8 (14.7 - 15)	14.9 (14.4 - 15.1)	10.9 (10.6 - 11.3)	15.3 (15 - 15.6)	16.7 (16 - 17)	14.9 (14.6 - 15)	13.9 (13.4 - 14.4)	19.1 (18.4 - 19.6)

Table S4. Region-specific results, tiered restrictions only. Burdens are summed over the period from 1 Oct 2020 to 31 March 2021. Weeks of high ICU occupancy is calculated by measuring the number of weeks in each region where ICU occupancy is 50% or greater than the peak occupancy during the first wave. Lockdowns are assumed to run from 5 November – 2 December 2020 inclusively. Medians and 95% projection intervals shown.

Indicator	England	East of England	London	Midlands	North East and Yorkshire	North West	South East	South West
Admissions	238,000 (231,000 - 245,000)	22,400 (21,100 - 23,500)	37,700 (35,300 - 41,000)	44,500 (42,500 - 48,600)	43,900 (39,300 - 47,500)	38,800 (37,300 - 41,000)	28,600 (26,900 - 31,600)	22,000 (20,000 - 23,500)
Deaths	48,600 (46,400 - 50,700)	5,360 (4,760 - 5,820)	4,020 (3,540 - 4,570)	9,430 (8,570 - 10,600)	10,600 (9,410 - 11,900)	8,260 (7,720 - 8,970)	5,880 (5,260 - 7,450)	4,900 (4,390 - 5,470)
Peak ICU (rel. W1)	131% (128 - 135%)	157% (147 - 175%)	80% (76 - 86%)	144% (136 - 151%)	205% (191 - 221%)	154% (147 - 170%)	119% (107 - 131%)	305% (276 - 344%)
Peak ICU requirement	3,900 (3,800 - 4,010)	426 (398 - 473)	926 (879 - 998)	629 (596 - 660)	635 (592 - 685)	550 (525 - 605)	419 (377 - 460)	298 (269 - 336)
Weeks in Tier 2	11.4 (10 - 12.7)	12 (7.85 - 12.6)	13.1 (10.4 - 16)	12.6 (9.43 - 16)	10 (5.71 - 10.7)	8.57 (4.57 - 9)	16 (9.43 - 16)	12.1 (7.71 - 13.1)
Weeks in Tier 3	4 (2.96 - 5.03)	4 (4 - 8)	4 (0 - 4)	4 (0 - 4)	4 (4 - 8)	4 (4 - 8)	0 (0 - 4)	4 (4 - 8)
Weeks in lockdown	0 (0 - 0)	0 (0 - 0)	0 (0 - 0)	0 (0 - 0)	0 (0 - 0)	0 (0 - 0)	0 (0 - 0)	0 (0 - 0)
Weeks of high ICU occupancy	14.6 (14.3 - 14.9)	14.9 (14.3 - 15.3)	9.57 (8.86 - 10.4)	14.9 (14.3 - 15.6)	17 (15.7 - 17.7)	14.7 (14 - 15.1)	13.3 (12.4 - 14.3)	20.1 (18.9 - 20.7)

Table S5. Region-specific results, Northern Ireland-type lockdown with schools open. Burdens are summed over the period from 1 Oct 2020 to 31 March 2021. Weeks of high ICU occupancy is calculated by measuring the number of weeks in each region where ICU occupancy is 50% or greater than the peak occupancy during the first wave. Lockdowns are assumed to run from 5 November – 2 December 2020 inclusively. Medians and 95% projection intervals shown.

Indicator	England	East of England	London	Midlands	North East and Yorkshire	North West	South East	South West
Admissions	206,000 (199,000 - 213,000)	20,300 (18,600 - 22,000)	33,400 (31,100 - 36,800)	37,100 (34,400 - 39,600)	37,700 (34,400 - 40,200)	32,900 (31,700 - 34,900)	24,200 (21,000 - 27,300)	20,300 (17,200 - 22,500)
Deaths	41,500 (39,600 - 43,400)	4,830 (4,190 - 5,320)	3,570 (3,160 - 4,090)	7,740 (6,950 - 8,490)	9,040 (7,980 - 9,960)	6,950 (6,440 - 7,690)	4,910 (4,060 - 5,970)	4,460 (3,670 - 5,110)
Peak ICU (rel. W1)	96% (93 - 102%)	102% (91 - 115%)	50% (45 - 57%)	98% (87 - 105%)	183% (173 - 196%)	152% (144 - 166%)	75% (61 - 88%)	226% (180 - 263%)
Peak ICU requirement	2,870 (2,760 - 3,040)	276 (246 - 313)	574 (516 - 660)	427 (378 - 459)	566 (534 - 606)	542 (511 - 591)	265 (215 - 309)	221 (176 - 256)
Weeks in Tier 2	12 (10.8 - 13.3)	13.9 (13.6 - 17.7)	17.5 (13.7 - 17.9)	11.1 (11 - 15.1)	6.29 (5.43 - 10.6)	0.714 (0.429 - 4.86)	15.7 (12.4 - 17.3)	17 (13.6 - 17.3)
Weeks in Tier 3	0.477 (0.368 - 0.575)	0 (0 - 0)	0 (0 - 0)	0 (0 - 0)	1 (0.429 - 1.71)	2.57 (2.14 - 2.71)	0 (0 - 0)	0 (0 - 0)
Weeks in lockdown	3.86 (3.86 - 3.86)	3.86 (3.86 - 3.86)	3.86 (3.86 - 3.86)	3.86 (3.86 - 3.86)	3.86 (3.86 - 3.86)	3.86 (3.86 - 3.86)	3.86 (3.86 - 3.86)	3.86 (3.86 - 3.86)
Weeks of high ICU occupancy	13.7 (12.9 - 14.7)	18.4 (17.7 - 19.2)	0 (0 - 6.44)	16.4 (14.7 - 17.4)	17.3 (15.7 - 18.3)	11.9 (11.1 - 12.3)	12.1 (8.71 - 17.1)	21.7 (21.4 - 22)

Table S6. Region-specific results, Northern Ireland-type lockdown with schools closed. Burdens are summed over the period from 1 Oct 2020 to 31 March 2021. Weeks of high ICU occupancy is calculated by measuring the number of weeks in each region where ICU occupancy is 50% or greater than the peak occupancy during the first wave. Lockdowns are assumed to run from 5 November – 2 December 2020 inclusively. Medians and 95% projection intervals shown.

Indicator	England	East of England	London	Midlands	North East and Yorkshire	North West	South East	South West
Admissions	177,000 (171,000 - 181,000)	16,700 (15,400 - 18,300)	29,200 (27,500 - 32,000)	31,700 (30,100 - 33,700)	31,800 (28,700 - 34,200)	28,800 (27,900 - 30,100)	20,100 (18,400 - 22,900)	18,400 (15,600 - 20,400)
Deaths	34,900 (33,500 - 36,700)	3,850 (3,380 - 4,250)	3,090 (2,700 - 3,500)	6,430 (5,950 - 7,200)	7,450 (6,600 - 8,350)	6,010 (5,620 - 6,580)	3,980 (3,440 - 4,700)	3,960 (3,360 - 4,500)
Peak ICU (rel. W1)	88% (85 - 91%)	83% (77 - 96%)	41% (38 - 48%)	92% (82 - 98%)	174% (165 - 188%)	150% (142 - 163%)	65% (56 - 77%)	208% (168 - 236%)
Peak ICU requirement	2,610 (2,520 - 2,720)	225 (209 - 261)	476 (441 - 553)	400 (360 - 427)	540 (509 - 581)	533 (506 - 580)	229 (198 - 270)	203 (164 - 230)
Weeks in Tier 2	8.48 (8.16 - 8.84)	13.9 (13.4 - 14.3)	13.9 (13.4 - 15.6)	3.14 (3.14 - 3.14)	2.14 (1.43 - 2.71)	0.571 (0.429 - 1)	12.7 (11.1 - 13.4)	15.1 (13 - 16.4)
Weeks in Tier 3	0.471 (0.358 - 0.574)	0 (0 - 0)	0 (0 - 0)	0 (0 - 0)	1 (0.429 - 1.71)	2.57 (2.14 - 2.71)	0 (0 - 0)	0 (0 - 0)
Weeks in lockdown	3.86 (3.86 - 3.86)	3.86 (3.86 - 3.86)	3.86 (3.86 - 3.86)	3.86 (3.86 - 3.86)	3.86 (3.86 - 3.86)	3.86 (3.86 - 3.86)	3.86 (3.86 - 3.86)	3.86 (3.86 - 3.86)
Weeks of high ICU occupancy	9.45 (9.12 - 9.95)	14.9 (13.1 - 16.7)	0 (0 - 0)	7 (6.29 - 8.29)	10.9 (10.4 - 11.9)	10 (9.57 - 10.3)	8.14 (6.26 - 9.43)	21.7 (21.4 - 22)

Table S7. Region-specific results, Wales-type lockdown with schools open. Burdens are summed over the period from 1 Oct 2020 to 31 March 2021. Weeks of high ICU occupancy is calculated by measuring the number of weeks in each region where ICU occupancy is 50% or greater than the peak occupancy during the first wave. Lockdowns are assumed to run from 5 November – 2 December 2020 inclusively. Medians and 95% projection intervals shown.

Indicator	England	East of England	London	Midlands	North East and Yorkshire	North West	South East	South West
Admissions	186,000 (179,000 – 193,000)	18,500 (16,900 – 20,000)	31,100 (28,900 – 35,100)	32,700 (30,900 – 35,100)	34,500 (31,700 – 37,600)	29,900 (28,500 – 31,800)	20,700 (18,400 – 23,800)	17,900 (15,300 – 20,100)
Deaths	36,800 (34,900 – 38,800)	4,340 (3,760 – 4,760)	3,270 (2,870 – 3,790)	6,730 (6,040 – 7,390)	8,180 (7,230 – 9,190)	6,260 (5,860 – 6,940)	4,100 (3,540 – 4,910)	3,800 (3,040 – 4,400)
Peak ICU (rel. W1)	90% (85 – 94%)	88% (80 – 101%)	44% (40 – 52%)	91% (83 – 99%)	176% (166 – 192%)	150% (142 – 163%)	66% (57 – 79%)	208% (164 – 235%)
Peak ICU requirement	2,670 (2,540 – 2,810)	239 (217 – 273)	507 (462 – 601)	399 (363 – 434)	543 (512 – 593)	534 (506 – 579)	232 (201 – 278)	203 (160 – 229)
Weeks in Tier 2	8.95 (8.28 – 9.62)	14 (13.6 – 17.9)	16.6 (13.6 – 18)	3.14 (3.14 – 7.14)	2.14 (1.43 – 2.72)	0.571 (0.429 – 1)	12.9 (11.1 – 14.6)	14.4 (13 – 16)
Weeks in Tier 3	0.473 (0.354 – 0.565)	0 (0 – 0)	0 (0 – 0)	0 (0 – 0)	1 (0.429 – 1.71)	2.57 (2.14 – 2.71)	0 (0 – 0)	0 (0 – 0)
Weeks in lockdown	3.86 (3.86 – 3.86)	3.86 (3.86 – 3.86)	3.86 (3.86 – 3.86)	3.86 (3.86 – 3.86)	3.86 (3.86 – 3.86)	3.86 (3.86 – 3.86)	3.86 (3.86 – 3.86)	3.86 (3.86 – 3.86)
Weeks of high ICU occupancy	11.3 (10.3 – 12.7)	20.4 (17.3 – 20.7)	0 (0 – 3.3)	7.43 (6.43 – 15.3)	16.8 (12.1 – 19.9)	10.3 (10 – 10.9)	8.71 (6.71 – 10.4)	21.7 (21.4 – 22)

Table S8. Region-specific results, Wales-type lockdown with schools closed. Burdens are summed over the period from 1 Oct 2020 to 31 March 2021. Weeks of high ICU occupancy is calculated by measuring the number of weeks in each region where ICU occupancy is 50% or greater than the peak occupancy during the first wave. Lockdowns are assumed to run from 5 November – 2 December 2020 inclusively. Medians and 95% projection intervals shown.

Indicator	England	East of England	London	Midlands	North East and Yorkshire	North West	South East	South West
Admissions	157,000 (152,000 - 163,000)	15,000 (13,600 - 16,400)	27,300 (25,600 - 30,100)	26,800 (25,100 - 28,600)	28,300 (25,800 - 30,800)	26,500 (25,400 - 28,000)	17,500 (15,500 - 19,300)	15,500 (13,400 - 17,600)
Deaths	30,300 (29,000 - 31,900)	3,370 (2,930 - 3,780)	2,850 (2,490 - 3,250)	5,400 (4,840 - 5,940)	6,590 (5,840 - 7,430)	5,490 (5,170 - 6,020)	3,380 (2,890 - 3,920)	3,220 (2,770 - 3,820)
Peak ICU (rel. W1)	87% (83 - 91%)	84% (77 - 95%)	41% (37 - 47%)	89% (78 - 95%)	172% (163 - 187%)	149% (142 - 162%)	66% (54 - 76%)	207% (166 - 231%)
Peak ICU requirement	2,590 (2,480 - 2,710)	227 (208 - 259)	470 (432 - 538)	388 (342 - 414)	531 (505 - 579)	531 (507 - 576)	232 (192 - 267)	203 (162 - 226)
Weeks in Tier 2	7.46 (7.11 - 7.78)	12.1 (11.3 - 13.1)	13.6 (12.6 - 14)	3.14 (3.14 - 3.14)	2.14 (1.43 - 2.71)	0.571 (0.429 - 1)	10 (8.14 - 11.7)	11.7 (10.6 - 13.2)
Weeks in Tier 3	0.473 (0.346 - 0.575)	0 (0 - 0)	0 (0 - 0)	0 (0 - 0)	1 (0.429 - 1.71)	2.57 (2.14 - 2.71)	0 (0 - 0)	0 (0 - 0)
Weeks in lockdown	3.86 (3.86 - 3.86)	3.86 (3.86 - 3.86)	3.86 (3.86 - 3.86)	3.86 (3.86 - 3.86)	3.86 (3.86 - 3.86)	3.86 (3.86 - 3.86)	3.86 (3.86 - 3.86)	3.86 (3.86 - 3.86)
Weeks of high ICU occupancy	7.92 (7.51 - 8.38)	11.6 (10.1 - 13.3)	0 (0 - 0)	6 (5.43 - 6.44)	9.71 (9.43 - 10.1)	9.43 (9.14 - 9.71)	5.57 (3.71 - 6.86)	18.4 (16.7 - 22)

Table S9. Projected cumulative proportion of the population ever infected with SARS-CoV-2 (attack rate) in the absence of tiers or lockdown, up to 31 March 2021.

Region	Attack rate
East of England	38% (38 - 38%)
London	46% (46 - 46%)
Midlands	37% (37 - 37%)
North East and Yorkshire	40% (40 - 40%)
North West	41% (41 - 41%)
South East	36% (36 - 36%)
South West	35% (35 - 35%)

Working group authors and acknowledgements

CMMID COVID-19 Working Group

The following authors were part of the Centre for Mathematical Modelling of Infectious Disease 2019-nCoV working group. Each contributed in processing, cleaning and interpretation of data, interpreted findings, contributed to the manuscript, and approved the work for publication: Petra Klepac, Thibaut Jombart, Rachel Lowe, Carl A B Pearson, Megan Auzenbergs, Joel Hellewell, Kevin van Zandvoort, Gwenan M Knight, Yang Liu, Oliver Brady, Kaja Abbas, Sebastian Funk, Hamish P Gibbs, Katherine E. Atkins, Yung-Wai Desmond Chan, Anna M Foss, Akira Endo, Damien C Tully, Amy Gimma, Rosalind M Eggo, Alicia Rosello, Kiesha Prem, Naomi R Waterlow, Katharine Sherratt, Graham Medley, Alicia Showering, Fiona Yueqian Sun, Billy J Quilty, Samuel Clifford, Matthew Quaife, Sophie R Meakin, Jack Williams, James D Munday, Stefan Flasche, Nikos I Bosse, Emily S Nightingale, C Julian Villabona-Arenas, Simon R Procter, Sam Abbott, Adam J Kucharski, Georgia R Gore-Langton, Frank G Sandmann, David Simons.

The following funding sources are acknowledged as providing funding for the working group authors. BBSRC LIDP (BB/M009513/1: DS). This research was partly funded by the Bill & Melinda Gates Foundation (INV-001754: MQ; INV-003174: KP, YL; NTD Modelling Consortium OPP1184344: CABP, GFM; OPP1180644: SRP; OPP1183986: ESN; OPP1191821: MA). BMGF (OPP1157270: KA). DFID/Wellcome Trust (Epidemic Preparedness Coronavirus research programme 221303/Z/20/Z: CABP, KvZ). Elrha R2HC/UK DFID/Wellcome Trust/This research was partly funded by the National Institute for Health Research (NIHR) using UK aid from the UK Government to support global health research. The views expressed in this publication are those of the author(s) and not necessarily those of the NIHR or the UK Department of Health and Social Care (KvZ). ERC Starting Grant (#757699: MQ). This project has received funding from the European Union's Horizon 2020 research and innovation programme - project EpiPose (101003688: KP, PK, YL). This research was partly funded by the Global Challenges Research Fund (GCRF) project 'RECAP' managed through RCUK and ESRC (ES/P010873/1: AG, TJ). HDR UK (MR/S003975/1: RME). MRC (MR/N013638/1: NRW). Nakajima Foundation (AE). NIHR (16/136/46: BJQ; 16/137/109: BJQ, FYS, YL; Health Protection Research Unit for Modelling Methodology HPRU-2012-10096: TJ; NIHR200908: RME; NIHR200929: FGS; PR-OD-1017-20002: AR). Royal Society (Dorothy Hodgkin Fellowship: RL; RP\EA\180004: PK). UK DHSC/UK Aid/NIHR (ITCRZ 03010: HPG). UK MRC (LID DTP MR/N013638/1: GRGL; MC_PC_19065: AG, RME, SC, TJ, YL; MR/P014658/1: GMK). Authors of this research receive funding from UK Public Health Rapid Support Team funded by the United Kingdom Department of Health and Social Care (TJ). Wellcome Trust (206250/Z/17/Z: AJK; 206471/Z/17/Z: OJB; 208812/Z/17/Z: SC, SFlasche; 210758/Z/18/Z: JDM, JH, KS, NIB, SA, SFunk, SRM). No funding (AMF, AS, CJVA, DCT, JW, KEA, YWDC).

ISARIC4C Consortium Investigators

The ISARIC4C Consortium Investigators include: Consortium Lead Investigator: J Kenneth Baillie, Chief Investigator: Malcolm G Semple, Co-Lead Investigator: Peter JM Openshaw. ISARIC Clinical Coordinator: Gail Carson. Co-Investigators: Beatrice Alex, Benjamin Bach, Wendy S Barclay, Debby Bogaert, Meera Chand, Graham S Cooke, Annemarie B Docherty, Jake Dunning, Ana da Silva Filipe, Tom Fletcher, Christopher A Green, Ewen M Harrison, Julian A Hiscox, Antonia Ying Wai Ho, Peter W Horby, Samreen Ijaz, Saye Khoo, Paul

Klenerman, Andrew Law, Wei Shen Lim, Alexander J Mentzer, Laura Merson, Alison M Meynert, Mahdad Noursadeghi, Shona C Moore, Massimo Palmarini, William A Paxton, Georgios Pollakis, Nicholas Price, Andrew Rambaut, David L Robertson, Clark D Russell, Vanessa Sancho-Shimizu, Janet T Scott, Thushan de Silva, Louise Sigfrid, Tom Solomon, Shiranee Sriskandan, David Stuart, Charlotte Summers, Richard S Tedder, Emma C Thomson, AA Roger Thompson, Ryan S Thwaites, Lance CW Turtle, Maria Zambon. Project Managers: Hayley Hardwick, Chloe Donohue, Ruth Lyons, Fiona Griffiths, Wilna Oosthuyzen. Data Analysts: Lisa Norman, Riinu Pius, Tom M Drake, Cameron J Fairfield, Stephen Knight, Kenneth A Mclean, Derek Murphy, Catherine A Shaw. Data and Information System Managers: Jo Dalton, James Lee, Daniel Plotkin, Michelle Girvan, Egle Saviciute, Stephanie Roberts, Janet Harrison, Laura Marsh, Marie Connor, Sophie Halpin, Clare Jackson, Carrol Gamble. Data integration and presentation: Gary Leeming, Andrew Law, Murray Wham, Sara Clohisey, Ross Hendry, James Scott-Brown. Material Management: William Greenhalf, Victoria Shaw, Sarah McDonald. Patient engagement: Seán Keating Outbreak Laboratory Staff and Volunteers: Katie A. Ahmed, Jane A Armstrong, Milton Ashworth, Innocent G Asiimwe, Siddharth Bakshi, Samantha L Barlow, Laura Booth, Benjamin Brennan, Katie Bullock, Benjamin WA Catterall, Jordan J Clark, Emily A Clarke, Sarah Cole, Louise Cooper, Helen Cox, Christopher Davis, Oslem Dincarslan, Chris Dunn, Philip Dyer, Angela Elliott, Anthony Evans, Lorna Finch, Lewis WS Fisher, Terry Foster, Isabel Garcia-Dorival, Willliam Greenhalf, Philip Gunning, Catherine Hartley, Antonia Ho, Rebecca L Jensen, Christopher B Jones, Trevor R Jones, Shadia Khandaker, Katharine King, Robyn T. Kiy, Chrysa Koukorava, Annette Lake, Suzannah Lant, Diane Latawiec, L Lavelle-Langham, Daniella Lefteri, Lauren Lett, Lucia A Livoti, Maria Mancini, Sarah McDonald, Laurence McEvoy, John McLauchlan, Soeren Metelmann, Nahida S Miah, Joanna Middleton, Joyce Mitchell, Shona C Moore, Ellen G Murphy, Rebekah Penrice-Randal, Jack Pilgrim, Tessa Prince, Will Reynolds, P. Matthew Ridley, Debby Sales, Victoria E Shaw, Rebecca K Shears, Benjamin Small, Krishanthi S Subramaniam, Agnieska Szemiel, Aislynn Taggart, Jolanta Tanianis-Hughes, Jordan Thomas, Erwan Trochu, Libby van Tonder, Eve Wilcock, J. Eunice Zhang. Local Principal Investigators: Kayode Adeniji, Daniel Agranoff, Ken Agwuh, Dhiraj Ail, Ana Alegria, Brian Angus, Abdul Ashish, Dougal Atkinson, Shahedal Bari, Gavin Barlow, Stella Barnass, Nicholas Barrett, Christopher Bassford, David Baxter, Michael Beadsworth, Jolanta Bernatoniene, John Berridge, Nicola Best, Pieter Bothma, David Brealey, Robin Brittain-Long, Naomi Bulteel, Tom Burden, Andrew Burtenshaw, Vikki Caruth, David Chadwick, Duncan Chambler, Nigel Chee, Jenny Child, Srikanth Chukkambotla, Tom Clark, Paul Collini, Catherine Cosgrove, Jason Cupitt, Maria-Teresa Cutino-Moguel, Paul Dark, Chris Dawson, Samir Dervisevic, Phil Donnison, Sam Douthwaite, Ingrid DuRand, Ahilanadan Dushianthan, Tristan Dyer, Cariad Evans, Chi Eziefula, Chrisopher Fegan, Adam Finn, Duncan Fullerton, Sanjeev Garg, Sanjeev Garg, Atul Garg, Effrossyni Gkrania-Klotsas, Jo Godden, Arthur Goldsmith, Clive Graham, Elaine Hardy, Stuart Hartshorn, Daniel Harvey, Peter Havalda, Daniel B Hawcutt, Maria Hobrok, Luke Hodgson, Anil Hormis, Michael Jacobs, Susan Jain, Paul Jennings, Agilan Kaliappan, Vidya Kasipandian, Stephen Kegg, Michael Kelsey, Jason Kendall, Caroline Kerrison, Ian Kerslake, Oliver Koch, Gouri Koduri, George Koshy, Shondipon Laha, Steven Laird, Susan Larkin, Tamas Leiner, Patrick Lillie, James Limb, Vanessa Linnett, Jeff Little, Michael MacMahon, Emily MacNaughton, Ravish Mankregod, Huw Masson, Elijah Matovu, Katherine McCullough, Ruth McEwen, Manjula Meda, Gary Mills, Jane Minton, Mariyam Mirfenderesky, Kavya Mohandas, Quen Mok, James Moon, Elinoor Moore, Patrick Morgan, Craig Morris, Katherine Mortimore, Samuel Moses, Mbiye Mpenge, Rohinton Mulla, Michael Murphy, Megan Nagel, Thapas Nagarajan, Mark Nelson, Igor Otahal,

Mark Pais, Selva Panchatsharam, Hassan Paraiso, Brij Patel, Natalie Pattison, Justin Pepperell, Mark Peters, Mandeep Phull, Stefania Pintus, Jagtur Singh Pooni, Frank Post, David Price, Rachel Prout, Nikolas Rae, Henrik Reschreiter, Tim Reynolds, Neil Richardson, Mark Roberts, Devender Roberts, Alistair Rose, Guy Rousseau, Brendan Ryan, Taranprit Saluja, Aarti Shah, Prad Shanmuga, Anil Sharma, Anna Shawcross, Jeremy Sizer, Manu Shankar-Hari, Richard Smith, Catherine Snelson, Nick Spittle, Nikki Staines, Tom Stambach, Richard Stewart, Pradeep Subudhi, Tamas Szakmany, Kate Tatham, Jo Thomas, Chris Thompson, Robert Thompson, Ascanio Tridente, Darell Tupper-Carey, Mary Twagira, Andrew Ustianowski, Nick Vallotton, Lisa Vincent-Smith, Shico Visuvanathan, Alan Vuylsteke, Sam Waddy, Rachel Wake, Andrew Walden, Ingeborg Welters, Tony Whitehouse, Paul Whittaker, Ashley Whittington, Meme Wijesinghe, Martin Williams, Lawrence Wilson, Sarah Wilson, Stephen Winchester, Martin Wiselka, Adam Wolverson, Daniel G Wooton, Andrew Workman, Bryan Yates, and Peter Young.

This work is supported by grants from: the National Institute for Health Research (NIHR; award CO-CIN-01), the Medical Research Council (MRC; grant MC_PC_19059), and by the NIHR Health Protection Research Unit (HPRU) in Emerging and Zoonotic Infections at University of Liverpool in partnership with Public Health England (PHE), in collaboration with Liverpool School of Tropical Medicine and the University of Oxford (award 200907), NIHR HPRU in Respiratory Infections at Imperial College London with PHE (award 200927), Wellcome Trust and Department for International Development (DID; 215091/Z/18/Z), the Bill and Melinda Gates Foundation (OPP1209135), Liverpool Experimental Cancer Medicine Centre (grant reference C18616/A25153), NIHR Biomedical Research Centre at Imperial College London (IS-BRC-1215-20013), EU Platform for European Preparedness Against (Re-)emerging Epidemics (PREPARE; FP7 project 602525), and NIHR Clinical Research Network for providing infrastructure support for this research. The views expressed are those of the authors and not necessarily those of the Department of Health and Social Care, DID, NIHR, MRC, Wellcome Trust, or PHE.

This work uses data provided by patients and collected by the NHS as part of their care and support #DataSavesLives. We are extremely grateful to the 2,648 frontline NHS clinical and research staff and volunteer medical students, who collected this data in challenging circumstances; and the generosity of the participants and their families for their individual contributions in these difficult times. We also acknowledge the support of Jeremy J Farrar, Nahoko Shindo, Devika Dixit, Nipunie Rajapakse, Lyndsey Castle, Martha Buckley, Debbie Malden, Katherine Newell, Kwame O'Neill, Emmanuelle Denis, Claire Petersen, Scott Mullaney, Sue MacFarlane, Nicole Maziere, Julien Martinez, Oslem Dincarslan, and Annette Lake.

References

1 Google. COVID-19 Community Mobility Reports. https://www.google.com/covid19/mobility/ (accessed Nov 2, 2020).

2 Mossong J, Hens N, Jit M, *et al.* Social contacts and mixing patterns relevant to the spread of infectious diseases. *PLoS Med* 2008; **5**: e74.

3 Jarvis CI, Van Zandvoort K, Gimma A, *et al.* Quantifying the impact of physical distance measures on the transmission of COVID-19 in the UK. *BMC Med* 2020; **18**: 124.

4 Wood S, Wood MS. The mgcv package. *www r-project org* 2007. http://btr0xq.rz.uni-bayreuth.de/math/statlib/R/CRAN/doc/packages/mgcv.pdf.

5 Wood SN. Fast stable restricted maximum likelihood and marginal likelihood estimation of semiparametric generalized linear models. *J R Stat Soc Series B Stat Methodol* 2011; **73**: 3–36.

6 Kissler SM, Tedijanto C, Goldstein E, Grad YH, Lipsitch M. Projecting the transmission dynamics of SARS-CoV-2 through the postpandemic period. *Science* 2020; **368**: 860–8.

7 Ter Braak CJF. A Markov Chain Monte Carlo version of the genetic algorithm Differential Evolution: easy Bayesian computing for real parameter spaces. Statistics and Computing. 2006; **16**: 239–49.

8 Lauer SA, Grantz KH, Bi Q, *et al.* The Incubation Period of Coronavirus Disease 2019 (COVID-19) From Publicly Reported Confirmed Cases: Estimation and Application. *Ann Intern Med* 2020; **172**: 577–82.

9 Davies NG, Kucharski AJ, Eggo RM, Gimma A, Edmunds WJ, Centre for the Mathematical Modelling of Infectious Diseases COVID-19 working group. Effects of non-pharmaceutical interventions on COVID-19 cases, deaths, and demand for hospital services in the UK: a modelling study. *Lancet Public Health* 2020; **5**: e375–85.

10 Ferretti L, Ledda A, Wymant C, *et al.* The timing of COVID-19 transmission. Epidemiology. 2020; published online Sept 7.

11 Davies NG, Klepac P, Liu Y, *et al.* Age-dependent effects in the transmission and control of COVID-19 epidemics. *medRxiv* 2020; : 2020.03.24.20043018.

12 Salje H, Tran Kiem C, Lefrancq N, *et al.* Estimating the burden of SARS-CoV-2 in France. *Science* 2020; **369**: 208–11.

13 Levin AT, Hanage WP, Owusu-Boaitey N, Cochran KB, Walsh SP, Meyerowitz-Katz G. Assessing the Age Specificity of Infection Fatality Rates for COVID-19: Systematic Review, Meta-Analysis, and Public Policy Implications. *medRxiv* 2020; : 2020.07.23.20160895.

14 Docherty AB, Harrison EM, Green CA, *et al.* Features of 20 133 UK patients in hospital with covid-19 using the ISARIC WHO Clinical Characterisation Protocol: prospective observational cohort study. *BMJ* 2020; **369**. DOI:10.1136/bmj.m1985.

15 Li Q, Guan X, Wu P, *et al.* Early Transmission Dynamics in Wuhan, China, of Novel Coronavirus-Infected Pneumonia. *N Engl J Med* 2020; **382**: 1199–207.

16 Nishiura H, Linton NM, Akhmetzhanov AR. Serial interval of novel coronavirus (COVID-19) infections. *Int J Infect Dis* 2020; **93**: 284–6.

17 Bi Q, Wu Y, Mei S, *et al.* Epidemiology and transmission of COVID-19 in 391 cases and 1286 of their close contacts in Shenzhen, China: a retrospective cohort study. *Lancet Infect Dis* 2020; **20**: 911–9.

18 Population estimates for the UK, England and Wales, Scotland and Northern Ireland - Office for National Statistics. https://www.ons.gov.uk/peoplepopulationandcommunity/populationandmigration/populationestimates/bulletins/annualmidyearpopulationestimates/mid2018 (accessed March 29, 2020).

19 Scientific Pandemic Influenza Group on Modelling (SPI-M). 2013; published online March 21. https://www.gov.uk/government/groups/scientific-pandemic-influenza-subgroup-on-modelling (accessed Nov 10, 2020).
